# PDGFRA is a conserved HAND2 effector during early cardiac development

**DOI:** 10.1038/s44161-024-00574-1

**Published:** 2024-12-10

**Authors:** Yanli Xu, Rupal Gehlot, Samuel J. Capon, Marga Albu, Jonas Gretz, Joshua Bloomekatz, Kenny Mattonet, Dubravka Vucicevic, Sweta Talyan, Khrievono Kikhi, Stefan Günther, Mario Looso, Beth A. Firulli, Miloslav Sanda, Anthony B. Firulli, Scott Allen Lacadie, Deborah Yelon, Didier Y. R. Stainier

**Affiliations:** 1 Department of Developmental Genetics, Max Planck Institute for Heart and Lung Research, Bad Nauheim, Germany; 2 Division of Biological Sciences, University of California, San Diego, La Jolla, CA USA; 3 Max Delbrück Center for Molecular Medicine in the Helmholtz Association (MDC), Berlin Institute for Medical Systems Biology (BIMSB), Berlin, Germany; 4 Bioinformatics Core Unit (BCU), Max Planck Institute for Heart and Lung Research, Bad Nauheim, Germany; 5 Flow Cytometry Service Group, Max Planck Institute for Heart and Lung Research, Bad Nauheim, Germany; 6 Bioinformatics and Deep Sequencing Platform, Max Planck Institute for Heart and Lung Research, Bad Nauheim, Germany; 7 Herman B Wells Center for Pediatric Research, Departments of Pediatrics, Anatomy and Medical and Molecular Genetics, Indiana Medical School, Indianapolis, IN USA; 8 Biomolecular Mass Spectrometry, Max Planck Institute for Heart and Lung Research, Bad Nauheim, Germany; 9 Present Address: Department of Biology, University of Mississippi, University, MS USA

**Keywords:** Organogenesis, Development

## Abstract

The basic helix–loop–helix transcription factor HAND2 has multiple roles during vertebrate organogenesis, including cardiogenesis. However, much remains to be uncovered about its mechanism of action. Here, we show the generation of several *hand2* mutant alleles in zebrafish and demonstrate that dimerization-deficient mutants display the null phenotype but DNA-binding-deficient mutants do not. Rescue experiments with Hand2 variants using a newly identified *hand2* enhancer confirmed these observations. To identify Hand2 effectors critical for cardiogenesis, we analyzed the transcriptomes of *hand2* loss- and gain-of-function embryonic cardiomyocytes and tested the function of eight candidate genes in vivo; *pdgfra* was most effective in rescuing myocardial migration in *hand2* mutants. Accordingly, we identified a putative Hand2-binding region in the zebrafish *pdgfra* locus that is important for its expression. In addition, *Hand2* loss- and gain-of-function experiments in mouse embryonic stem cell-derived cardiac cells decreased and increased *Pdgfra* expression, respectively. Altogether, these results further our mechanistic understanding of HAND2 function during early cardiogenesis.

## Abstract (web summary)

Xu and colleagues show that the transcription factor Hand2 promotes *pdgfra* expression during early cardiogenesis and that it can do so independently of direct DNA binding by interacting with Tcf3.

## Main

In all vertebrates, cardiac precursors emerge and differentiate in the anterior lateral plate mesoderm (LPM)^1–3^. During this time, bilateral populations of myocardial precursors move toward the midline where they fuse and form a cardiac crescent or disc that elongates to form the embryonic heart tube^1^. The genetic and molecular regulation of these developmental processes is complex. Mutations in several genes encoding transcription factors involved in early cardiogenesis (including GATA-binding protein 4 (GATA4), GATA5, GATA6, NK2 homeobox 5 (NKX2.5), heart- and neural crest derivatives expressed 1 (HAND1) and HAND2) result in diverse types of congenital heart defects^4–11^. Therefore, investigating the biological processes and mechanisms that govern vertebrate heart development will enhance our understanding of human cardiac malformations and diseases.

The basic helix–loop–helix (bHLH) transcription factor HAND2 is expressed in the LPM of zebrafish^12–14^, frog^12^, chick^15^ and mouse embryos^15^ and has multiple critical roles during cardiac development. In mammals, there are two HAND proteins: HAND1 and HAND2; they function as homodimers or heterodimers, binding to consensus E-box (CANNTG) or D-box (CGNNTG) motifs within the regulatory regions of target genes to control their expression^16,17^. *Hand1* and *Hand2* exhibit partially overlapping expression patterns in the developing mouse heart^18–20^. While *Hand2* mutants display right ventricular hypoplasia and vascular malformations^21,22^, *Hand1* global loss-of-function mutants die by embryonic day 9.5 (E9.5) owing to defects in extraembryonic tissue and heart morphogenesis, with the latter defects arising from impaired heart tube formation^23,24^. Previous genetic studies have shown that a DNA-binding-defective mutant of HAND2 is as effective as the wild-type (WT) protein for early mouse heart development^25^; however, loss- and gain-of-function studies in the mouse limb have revealed the requirement for the DNA-binding domain of HAND2 in that tissue^25,26^. These and other findings^25–27^ suggest that HAND2 regulates tissue growth and development through DNA-binding-dependent and -independent mechanisms.

Studies in zebrafish can provide additional clues regarding the function of HAND during cardiac development. In some teleosts, including zebrafish, only a single *hand* gene, *hand2*, has been reported^14^, which has helped investigate Hand function; however, other teleosts (for example, medaka) have two *hand* genes: *hand1* and *hand2* (ref. ^28^). Zebrafish *hand2* mutants display cardia bifida, a phenotype whereby the bilateral populations of myocardial precursors fail to fuse in the midline^14^. In addition, zebrafish *hand2* mutants exhibit myocardial differentiation defects^14,29^. These mouse and zebrafish studies raise questions about the precise mechanism of action of HAND proteins and the identity of the HAND2 effector genes that drive cardiac morphogenesis. Here, we generated dimerization-deficient and DNA-binding-deficient mutant alleles of *hand2* in zebrafish. We found that, as in mice, the DNA-binding domain of Hand2 is dispensable during early cardiogenesis in zebrafish, but that its dimerization domain is required. Transcriptomic studies followed by an in vivo phenotypic rescue assay identified *pdgfra* as an effector of Hand2 during early cardiogenesis in zebrafish. We further found that HAND2 regulates *Pdgfra* expression in cardiac precursors in mice and in mouse embryonic stem cell (mESC)-derived cardiac cells. Altogether, these results indicate that *Pdgfra* is a conserved effector of HAND2 during early cardiogenesis.

## Results

### The DNA-binding domain of Hand2 is not required for early zebrafish cardiogenesis

**Fig. 1:**
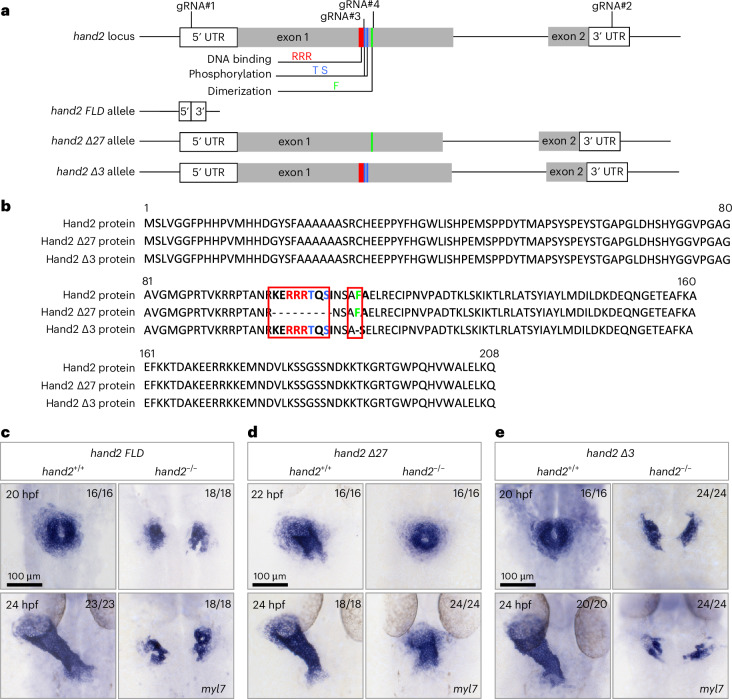
The DNA-binding domain of Hand2 is not required for early zebrafish cardiogenesis. **a**, Schematic of the *hand2* locus and *hand2* mutants. A *hand2 FLD* allele was generated by targeting the 5′ and 3′ UTR sequences of *hand2* (gRNA#1, gRNA#2), resulting in a deletion of 1,411 bp. A *hand2* DNA-binding and phosphorylation domains-deficient allele was generated using one gRNA (gRNA#3) around the sequence encoding the DNA-binding and phosphorylation domains of Hand2, resulting in a 27-bp deletion. A *hand2* dimerization domain-deficient allele was generated using one gRNA (gRNA#4) around the sequence encoding the dimerization domain of Hand2, resulting in a 3-bp deletion. **b**, Amino acid sequence alignment of Hand2, Hand2 Δ27 and Hand2 Δ3 generated through Clustal Omega. The three arginines in red and the threonine and serine in blue were deleted in the DNA-binding and phosphorylation domains-deficient form of Hand2 (Hand2 Δ27); the phenylalanine in green was deleted in the dimerization domain-deficient form of Hand2 (Hand2 Δ3). **c**, In situ hybridization showing *myl7* expression in 20- and 24-hpf *hand2 FLD*^+/+^ and *hand2 FLD*^−/−^ sibling embryos. **d**, In situ hybridization showing *myl7* expression in 22- and 24-hpf *hand2 Δ27*^+/+^ and *hand2 Δ27*^−/−^ sibling embryos. **e**, In situ hybridization showing *myl7* expression in 20- and 24-hpf *hand2 Δ3*^+/+^ and *hand2 Δ3*^−/−^ sibling embryos. The proportion of embryos matching the image shown is indicated in the top right corner of each image. The scale bars apply to all images.

**Extended Data Fig. 1:**
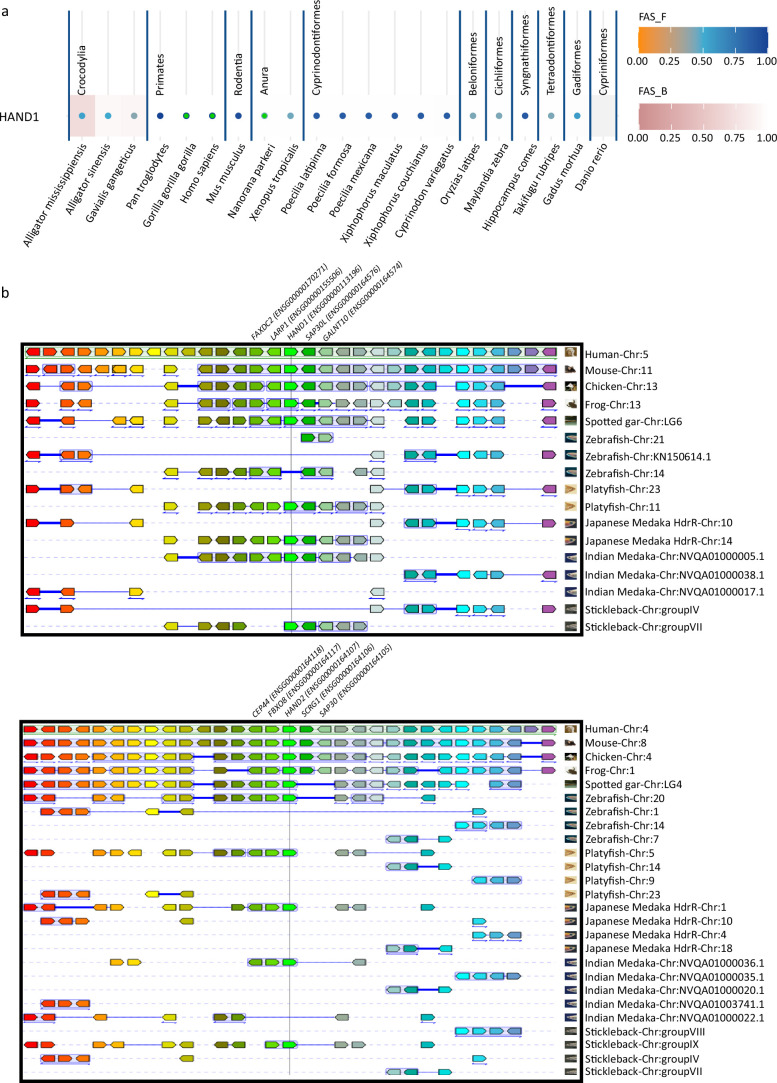
Loss of *hand1* in zebrafish. **a**, Phylogenetic profile of HAND1 on a species level resolution. Colour code indicates the feature architecture similarity (FAS) score of the orthologues to the human seed protein. Dot colour (FAS_F) and cell colour (FAS_B) represent the feature architecture similarity score between two orthologs using the human protein (dot colour) and the ortholog (cell colour) as reference, respectively. **b**, PhyloView representation of HAND1 and HAND2 in the human genome and their ortholog in other genomes. HAND1/HAND2 and their orthologs are positioned in the center, aligned with their neighboring genes, in various genomes. Genes of the same color represent orthologs.

**Extended Data Fig. 2:**
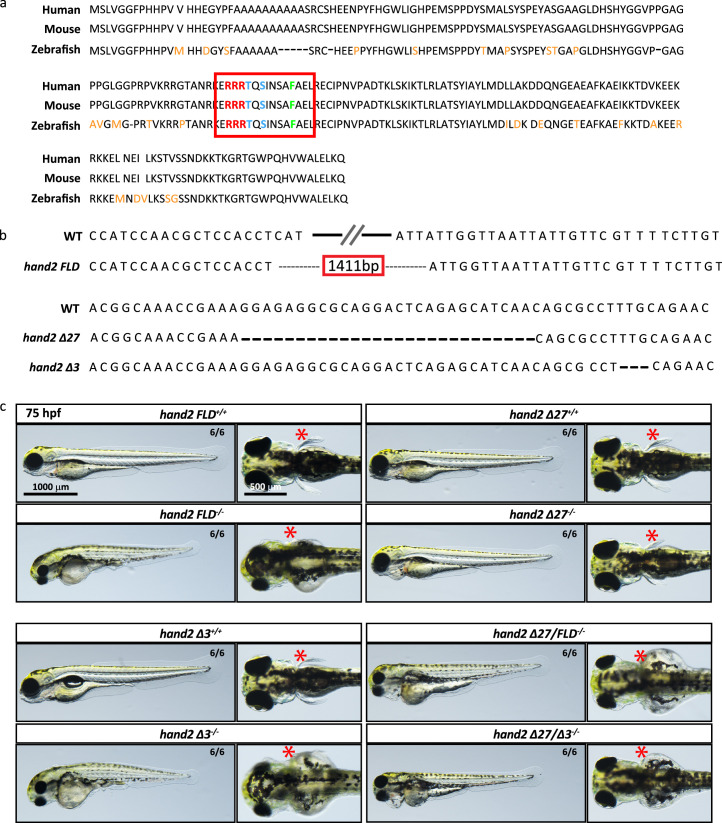
Generation of *hand2* mutant alleles. **a**, Amino acid sequence alignment of Human HAND2, mouse HAND2, and zebrafish Hand2 generated through Clustal Omega. The three arginines in red have DNA binding activity; the threonine and serine in blue have phosphorylation activity; the phenylalanine in green has dimerization activity. While there is 100% amino acid sequence identity between human and mouse HAND2, the residues that are different in zebrafish are indicated in orange. **b**, Partial nucleotide sequence of the *hand2 FLD* allele, which shows a 1,411 bp deletion, the *hand2 Δ27* allele, and the *hand2 Δ3* allele. **c**, Brightfield images of 75 hpf *hand2 FLD*^*+/+*^ and *hand2 FLD*^−*/*−^ sibling larvae, *hand2 Δ27*^*+/+*^ and *hand2 Δ27*^−*/*−^ sibling larvae, *hand2 Δ3*^*+/+*^ and *hand2 Δ3*^−*/*−^ sibling larvae, *hand2 Δ27/FLD* transheterozygous larvae, and *hand2 Δ27/Δ3* transheterozygous larvae. The proportion of larvae matching the image shown is indicated in the top right corner of each image. Scale bars apply to all images.

Previous studies have shown that, in some contexts, HAND2 can function independently of direct DNA binding. For example, mice in which the *Hand2* gene was replaced with a DNA-binding-deficient variant exhibited relatively normal hearts at E11.5 (ref. ^25^), in contrast with the severe ventricular hypoplasia observed in *Hand2* mutants at E10.5 (ref. ^22^). It is hypothesized that the DNA-binding-deficient variant of HAND2 can influence transcription through dimerization with other bHLH factors such as HAND1, as well as through interactions with large protein complexes^15,16,30–35^. In support of this hypothesis, another study has shown that overexpression of *hand2* in early zebrafish embryos enhances cardiomyocyte production and that this activity is dependent on the Hand2 dimerization domain but not its DNA-binding domain^27^. We first investigated the evolutionary history of *HAND1* with a particular focus on its fate in some fish, amphibian, reptile and mammalian species. Phylogenetic profiling revealed that *Danio rerio* (zebrafish) does not have an annotated *hand1* gene (Extended Data Fig. 1a). To increase the resolution of this comparative analysis further, we investigated the gene neighborhood around *HAND1* across vertebrates and found that the neighboring genes are all present in zebrafish (Extended Data Fig. 1b). We also examined the gene neighborhood around *HAND2* across vertebrates and found that it is conserved (Extended Data Fig. 1b). Together, these data indicate that zebrafish, unlike some other teleosts, have only one *hand* gene, which corresponds to *hand2*. In this context, we wanted to test whether the role of Hand2 during early cardiac development in zebrafish was dependent on its DNA-binding and/or dimerization domains, which are easily identified as the HAND2 bHLH domain is highly conserved (Extended Data Fig. 2a). To this end, we first generated a *hand2* full-locus deletion allele (*hand2 FLD*^*bns539*^) (that is, a null allele), a *hand2* DNA-binding and phosphorylation domains-deficient allele (*hand2 Δ27*^*bns540*^) and a *hand2* dimerization domain-deficient allele (*hand2 Δ3*^*bns603*^) (Fig. 1a). The *hand2 FLD* allele has a 1,411-base-pair (bp) deletion that spans the entire *hand2* locus (Extended Data Fig. 2b). To generate an allele deficient in the DNA-binding and phosphorylation domains and an allele deficient in the dimerization domain, we used guide RNAs (gRNAs) around these regions, resulting in a 27-bp deletion and a 3-bp deletion, respectively (Extended Data Fig. 2b). The mutation in the DNA-binding and phosphorylation domains led to a nine-amino-acid deletion in the highly conserved DNA-binding and phosphorylation region (Fig. 1b); the dimerization mutation led to the deletion of a phenylalanine (Fig. 1b) that has been shown to be necessary for dimerization^26^.

Consistent with previous reports on zebrafish *hand2* mutants^14,36,37^, *hand2 FLD*^−/−^ embryos displayed the characteristic cardia bifida phenotype (Fig. 1c) as well as pectoral fin defects (Extended Data Fig. 2c). In contrast, cardiac development proceeded normally in *hand2 Δ27*^−/−^ embryos, although with a slight delay (Fig. 1d), and mild defects in pectoral fin formation were observed (Extended Data Fig. 2c). However, *hand2 Δ3*^−/−^ embryos display the null phenotype (Fig. 1e). *hand2 Δ27/Δ3* transheterozygous embryos display a more pronounced pericardial edema and more severe pectoral fin defects than *hand2 Δ27*^−/−^ embryos (Extended Data Fig. 2c). Genotyping of phenotypically WT, 10 dpf (days after fertilization) larvae from *hand2 Δ27*^+/−^ intercrosses revealed 31 *hand2 Δ27*^−/−^ offspring among 151 examined; however, we did not find *hand2 Δ27*^−/−^ offspring at 15 dpf or 4 months of age, indicating that the DNA-binding domain of Hand2, while not needed for embryonic or early larval development, is required for survival past the midlarval stages. Altogether, these results indicate that Hand2 dimerization, but not its DNA-binding capability, is required for early cardiac development.

**Extended Data Fig. 3:**
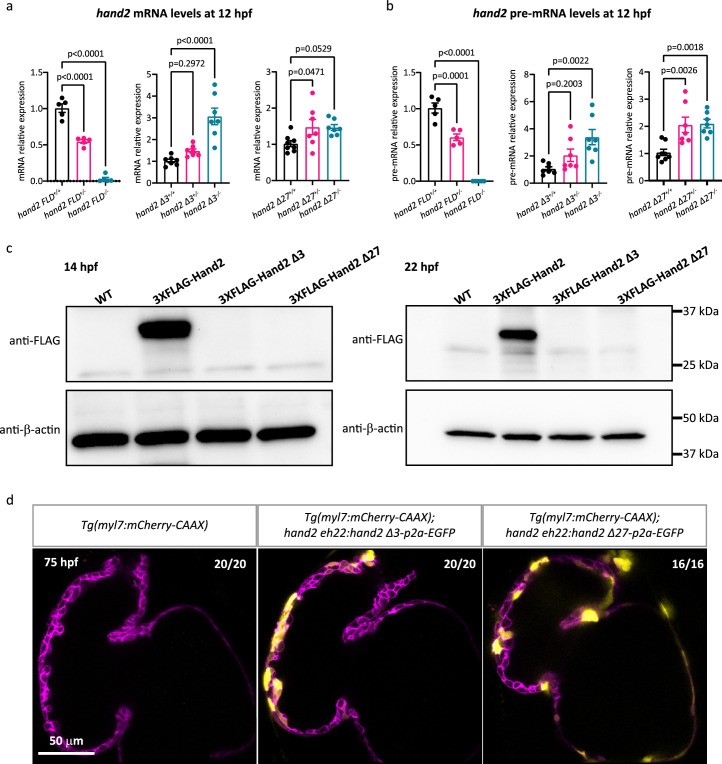
*hand2* expression in *hand2* mutant alleles. **a**, Relative mRNA levels of *hand2* in 12 hpf *hand2 FLD*^*+/+*^, *hand2 FLD*^*+/*−^, and *hand2 FLD*^−*/*−^ sibling embryos; relative mRNA levels of *hand2* in 12 hpf *hand2 Δ3*^*+/+*^, *hand2 Δ3*^*+/*−^, and *hand2 Δ3*^−*/*−^ sibling embryos; relative mRNA levels of *hand2* in 12 hpf *hand2 Δ27*^*+/+*^, *hand2 Δ27*^*+/*−^, and *hand2 Δ27*^−*/*−^ sibling embryos; error bars are mean ± s.e.m; *n* = 5 *hand2 FLD*^*+/+*^, *n* = 5 *hand2 FLD*^*+/*−^, *n* = 5 *hand2 FLD*^−*/*−^; *n* = 7 *hand2 Δ3*^*+/+*^, *n* = 7 *hand2 Δ3*^*+/*−^, *n* = 7 *hand2 Δ3*^−*/*−^; *n* = 8 *hand2 Δ27*^*+/+*^, *n* = 7 *hand2 Δ27*^*+/*−^, *n* = 7 *hand2 Δ27*^−*/*−^. **b**, Relative pre-mRNA levels of *hand2* in 12 hpf *hand2 FLD*^*+/+*^, *hand2 FLD*^*+/*−^, and *hand2 FLD*^−*/*−^ sibling embryos; relative pre-mRNA levels of *hand2* in 12 hpf *hand2 Δ3*^*+/+*^, *hand2 Δ3*^*+/*−^, and *hand2 Δ3*^−*/*−^ sibling embryos; relative pre-mRNA levels of *hand2* in 12 hpf *hand2 Δ27*^*+/+*^, *hand2 Δ27*^*+/*−^, and *hand2 Δ27*^−*/*−^ sibling embryos; error bars are mean ± s.e.m; *n* = 5 *hand2 FLD*^*+/+*^, *n* = 5 *hand2 FLD*^*+/*−^, *n* = 5 *hand2 FLD*^−*/*−^; *n* = 7 *hand2 Δ3*^*+/+*^, *n* = 7 *hand2 Δ3*^*+/*−^, *n* = 7 *hand2 Δ3*^−*/*−^; *n* = 8 *hand2 Δ27*^*+/+*^, *n* = 7 *hand2 Δ27*^*+/*−^, *n* = 7 *hand2 Δ27*^−*/*−^. **c**, Western blot analysis comparing the levels of FLAG-tagged Hand2 protein in 14 and 22 hpf *3xFLAG-hand2*, *3xFLAG-hand2 Δ3*, and *3xFLAG-hand2 Δ27* mRNA injected embryos. **d**, Confocal images of representative *Tg(myl7:mCherry-CAAX)* hearts from 72 hpf larvae that were not injected, or injected at the one-cell stage with a *hand2 eh22:hand2 Δ3-p2a-EGFP* plasmid, or a *hand2 eh22:hand2 Δ27-p2a-EGFP* plasmid. *P* values in a, b were calculated using a one-way ANOVA multiple comparison test. The proportion of larvae matching the image shown is indicated in the top right corner of each image. Scale bar applies to all images. C_t_ values of qPCR data are listed in Supplementary Table 1.

To investigate the reason for the differences between the *hand2 Δ27* and *Δ3* mutant phenotypes at a molecular level, we first examined *hand2* mRNA and pre-mRNA levels at 12 hpf (hours after fertilization). qPCR analysis showed that *hand2 Δ27*^−/−^ and *Δ3*^−/−^ embryos exhibit increased *hand2* expression at 12 hpf, the time of initial *hand2* expression (Extended Data Fig. 3a,b), suggesting that the early embryonic phenotypes in *hand2 Δ3* mutants are not due to reduced *hand2* mRNA or pre-mRNA levels. We also examined the stability of Hand2 Δ27 and Δ3 by western blot analysis of proteins exogenously expressed in zebrafish embryos and found that they were both less stable than the WT (Extended Data Fig. 3c). Thus, while both the Δ27 and Δ3 deletions affect Hand2 protein stability, only the Δ3 deletion leads to an early cardiac phenotype, indicating that protein instability is not the main reason for the phenotypic differences between the *hand2 Δ27* and *Δ3* mutants. To examine further the function of Hand2 Δ27 and Δ3 proteins and test whether they may function as dominant negatives, we overexpressed them in WT embryos; however, we found that they did not cause cardia bifida (Extended Data Fig. 3d), consistent with the fact that we did not observe obvious cardiac phenotypes in *hand2 Δ27*^+/−^ or *Δ3*^+/−^ embryos.

### Cardiac fusion requires the dimerization domain of Hand2

**Extended Data Fig. 4:**
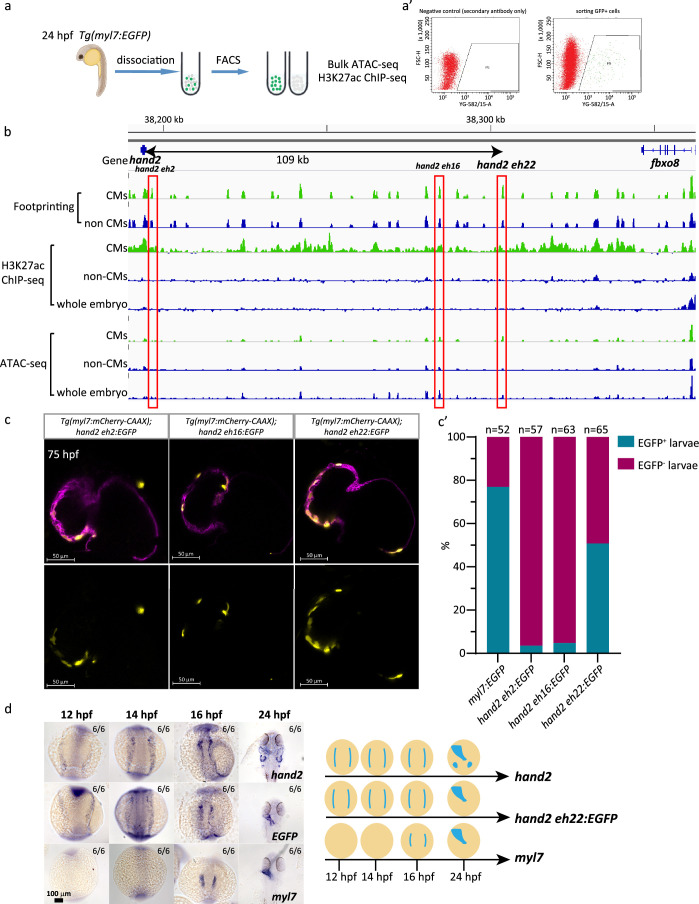
*hand2 eh22* enhancer-driven reporter labels early cardiac precursors. **a**, Schematic representation of ATAC-seq and H3K27ac ChIP-seq experimental design; a *Tg(myl7:EGFP)* line was used to isolate EGFP^+^ (that is, myocardial) cells and EGFP^−^ (that is, non-myocardial) cells at 24 hpf. a’, Graphs showing the FACS gating strategies to sort 24 hpf *Tg(myl7:*EGFP + *)* CMs. **b**, Genome browser view showing ATAC-seq and ChIP-seq peaks enriched in myocardial cells at the *hand2* locus; red boxes indicate 3 of the putative enhancers that were tested. **c**, Confocal images of representative *Tg(myl7:mCherry-CAAX)* hearts from 75 hpf larvae that were injected at the one-cell stage with a *hand2 eh2:EGFP, hand2 eh16:EGFP*, or *hand2 eh22:EGFP* plasmid. c’, Percentage of EGFP+ embryos that were injected at the one-cell stage with a *myl7:EGFP, hand2 eh2:EGFP, hand2 eh16:EGFP*, or *hand2 eh22:EGFP* plasmid. **d**, In situ hybridization showing *hand2*, *EGFP*, and *myl7* expression in 12, 14, 16, and 24 hpf WT and *Tg(hand2 eh22:EGFP)* sibling embryos; schematics of the expression pattern shown on the right. The proportion of embryos matching the image shown is indicated in the top right corner of each image. Scale bar applies to all images. Panel **a** created with BioRender.com.

The gene *hand2* is broadly expressed within the LPM^14^, which gives rise to various cell lineages and tissues, including the cardiovascular system, blood, mesothelium and limb connective tissue^38^. Additionally, at later developmental stages, *hand2* reporter-expressing cells can be detected in the heart, as well as in endoderm-derived organs such as the liver and pancreas^13^. To investigate further the requirement for Hand2 in the early events of cardiac lineage specification in zebrafish, we next characterized the *hand2*-expressing early cardiac precursors. The myosin light chain 7 gene (*myl7*) is one of the earliest markers of the myocardial lineage and is expressed in the anterior LPM by the 13-somite stage (15 hpf) and subsequently in differentiated cardiomyocytes in both chambers^39,40^. We isolated cardiomyocytes from 24 hpf *Tg(myl7:EGFP)* embryos using fluorescence-activated cell sorting (FACS) and performed bulk ATAC–seq (assay for transposase-accessible chromatin followed by sequencing) and H3K27ac ChIP–seq (chromatin immunoprecipitation followed by sequencing) on them (Extended Data Fig. 4a). We integrated these ATAC–seq and H3K27ac ChIP–seq data to perform a cardiomyocyte footprinting analysis^41^ and identified 22 enriched open chromatin regions upstream of the *hand2* transcription start site in *myl7* reporter-expressing cells (Extended Data Fig. 4b) that contained transcription factor-binding motifs. To identify a *hand2* enhancer that can drive *EGFP* expression in cardiac precursors, we cloned each of these 22 open chromatin regions to generate independent *hand2 enhancer:EGFP* plasmids, injected each of them into one-cell-stage embryos, and examined the expression pattern of EGFP at 24 and 48 hpf. Notably, we observed a specific EGFP signal in the cardiomyocytes of a majority (33 of 65) of the injected embryos only when testing a particular 752-bp enhancer (*hand2 eh22*), whereas the other potential enhancers (for example, *hand2 eh2* and *eh16*) drove EGFP expression in the cardiomyocytes of a minority (2 of 57 and 3 of 63, respectively) of the injected embryos (Extended Data Fig. 4b,c′). This *hand2 eh22* enhancer-driven EGFP expression is much more specific for cardiomyocytes than the broad expression observed in the *TgBAC(hand2:EGFP)*^*pd24*^ line, which recapitulates endogenous *hand2* expression in multiple tissues^13^.

**Fig. 2:**
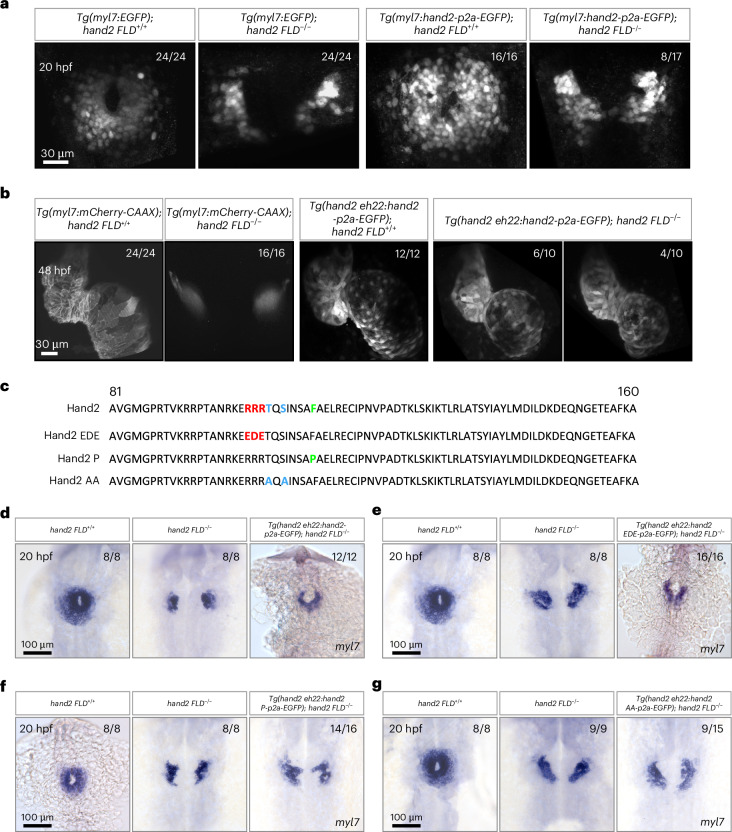
Cardiac fusion requires the dimerization domain of Hand2. **a**, Maximum intensity projections of confocal images of *Tg*(*myl7*:EGFP) expression in 20 hpf *hand2 FLD*^+/+^ and *hand2 FLD*^−/−^ sibling embryos, as well as *Tg*(*myl7*:hand2-p2a-EGFP) expression in 20 hpf *hand2 FLD*^+/+^ and *hand2 FLD*^−/−^ sibling embryos (of the 17 *Tg(myl7:hand2-p2a-EGFP)*; *hand2 FLD*^−/−^ embryos, 9 displayed no rescue of myocardial migration and 8 displayed partial rescue). **b**, Maximum intensity projections of confocal images of *Tg*(*myl7*:mCherry-CAAX) expression in 48 hpf *hand2 FLD*^+/+^ and *hand2 FLD*^−/−^ sibling embryos, as well as *Tg(hand2 eh22:*hand2-p2a-EGFP) expression in 48 hpf *hand2 FLD*^+/+^ and *hand2 FLD*^−/−^ sibling embryos. **c**, Partial amino acid sequence alignment of Hand2, Hand2 EDE, Hand2 P and Hand2 AA. EDE: DNA-binding-deficient form of Hand2 (RRR to EDE); P: dimerization-deficient form of Hand2 (F to P); AA: phosphorylation-deficient form of Hand2 (TS to AA). **d**, In situ hybridization showing *myl7* expression in 20 hpf *hand2 FLD*^+/+^, *hand2 FLD*^−/−^ and *Tg(hand2 eh22:hand2-p2a-EGFP)*; *hand2 FLD*^−/−^ sibling embryos. **e**, In situ hybridization showing *myl7* expression in 20 hpf *hand2 FLD*^+/+^, *hand2 FLD*^−/−^ and *Tg(hand2 eh22:hand2 EDE-p2a-EGFP)*; *hand2 FLD*^−/−^ sibling embryos. **f**, In situ hybridization showing *myl7* expression in 20 hpf *hand2 FLD*^+/+^, *hand2 FLD*^−/−^ and *Tg(hand2 eh22:hand2 P-p2a-EGFP)*; *hand2 FLD*^−/−^ sibling embryos (of the 16 *Tg(hand2 eh22:hand2 P-p2a-EGFP)*; *hand2 FLD*^−/−^ embryos, 14 displayed no rescue of myocardial migration and 2 displayed WT *hand2*-like rescue). **g**, In situ hybridization showing *myl7* expression in 20 hpf *hand2 FLD*^+/+^, *hand2 FLD*^−/−^ and *Tg(hand2 eh22:hand2 AA-p2a-EGFP)*; *hand2 FLD*^−/−^ sibling embryos (of the 15 *Tg(hand2 eh22:hand2 AA-p2a-EGFP)*; *hand2 FLD*^−/−^ embryos, 9 displayed no rescue of myocardial migration and 6 displayed WT *hand2*-like rescue). All embryos are shown in dorsal views, anterior to the top. The proportion of embryos matching the image shown is indicated in the top right corner of each image. The scale bars apply to all images.

To determine whether the *hand2 eh22* enhancer could be used to label early cardiac precursors, we generated a *Tg(hand2 eh22:EGFP)*^*bns623*^ line. Owing to the lag time between *EGFP* transcription and EGFP protein accumulation, we performed in situ hybridization using an *EGFP* probe to detect enhancer activity at early developmental stages (that is, 12, 14, 16 and 24 hpf). We observed *EGFP* mRNA expression as early as 12 hpf (Extended Data Fig. 4d), which coincides with the start of expression of endogenous *hand2*. Notably, the *hand2 eh22* enhancer-driven *EGFP* expression appeared earlier than *myl7* expression (Extended Data Fig. 4d). To elucidate further the functional properties of this enhancer, we generated a line that expresses *hand2* under the *myl7* promoter, *Tg(myl7:hand2-p2a-EGFP)*^*bns610*^, and another line that expresses *hand2* under the *hand2 eh22* enhancer, *Tg(hand2 eh22:hand2-p2a-EGFP)*^*bns665*^. Interestingly, there was only a partial rescue of myocardial migration in some (8 of 17) *Tg(myl7:hand2-p2a-EGFP)*; *hand2 FLD*^−/−^ embryos at 20 hpf (Fig. 2a) and no rescue in the other 9 embryos; however, overexpression of *hand2* under the *hand2 eh22* enhancer led to a complete rescue of the cardia bifida phenotype in *hand2 FLD*^−/−^ animals, as well as a looped and beating heart at 48 hpf (Fig. 2b).

**Extended Data Fig. 5:**
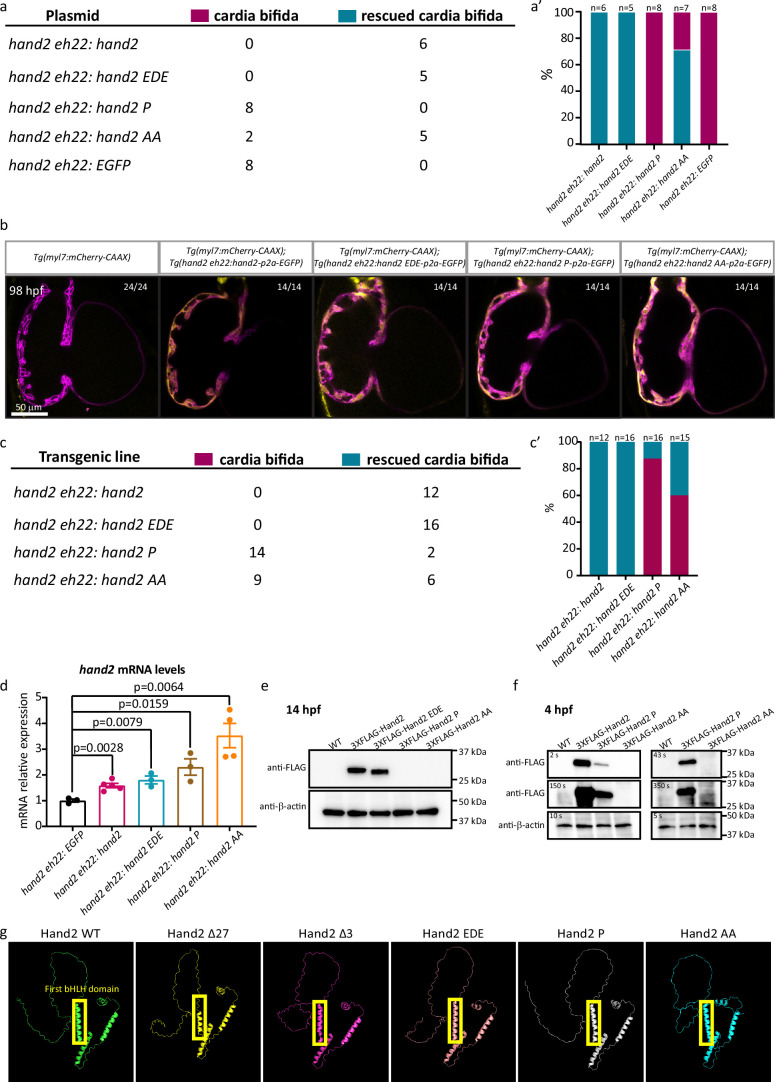
*hand2* expression in *hand2* OE lines. **a**, Proportion (a) and percentage (a’) of rescued 48 hpf *hand2 FLD*^−*/*−^ embryos after injection at the one-cell stage with the constructs as listed (as in Fig. 2b). **b**, Confocal images of representative *Tg(myl7:mCherry-CAAX)* hearts from 98 hpf *Tg(hand2 eh22:hand2-p2a-EGFP)*, *Tg(hand2 eh22:hand2 EDE-p2a-EGFP), Tg(hand2 eh22:hand2 P-p2a-EGFP)*, and *Tg(hand2 eh22:hand2 AA-p2a-EGFP)* larvae. **c**, Proportion (c) and percentage (c’) of rescued 20 hpf *Tg(hand2 eh22:hand2-p2a-EGFP)*;*hand2 FLD*^−*/*−^, *Tg(hand2 eh22:hand2 EDE-p2a-EGFP)*;*hand2 FLD*^−*/*−^, *Tg(hand2 eh22:hand2 P-p2a-EGFP)*;*hand2 FLD*^−*/*−^, and *Tg(hand2 eh22:hand2 AA-p2a-EGFP)*;*hand2 FLD*^−*/*−^ embryos. **d**, Relative mRNA levels of *hand2* in 20 hpf *Tg(hand2 eh22:EGFP), Tg(hand2 eh22:hand2-p2a-EGFP)*, *Tg(hand2 eh22:hand2 EDE-p2a-EGFP), Tg(hand2 eh22:hand2 P-p2a-EGFP)*, and *Tg(hand2 eh22:hand2 AA-p2a-EGFP)* EGFP^+^ cells; error bars are mean ± s.e.m.; *n* = 3 *hand2 eh22:EGFP*, *n* = 5 *hand2 eh22:hand2*, *n* = 3 *hand2 eh22:hand2 EDE*, *n* = 3 *hand2 eh22:hand2 P*, *n* = 4 *hand2 eh22:hand2 AA*. **e**, Western blot analysis comparing the levels of FLAG-tagged Hand2 protein in 14 hpf *3xFLAG-hand2*, *3xFLAG-hand2 EDE*, *3xFLAG-hand2 P*, and *3xFLAG-hand2 AA* mRNA injected embryos. **f**, Western blot analysis comparing the levels of FLAG-tagged Hand2 protein in 4 hpf *3xFLAG-hand2*, *3xFLAG-hand2 P*, and *3xFLAG-hand2 AA* mRNA injected embryos with different exposure times for protein detection. **g**, Modeling of the structure of Hand2, Hand2 Δ27, Hand2 Δ3, Hand2 EDE, Hand2 P and Hand2 AA by Alphafold2. *P* values in d were calculated using a one-way ANOVA multiple comparison test. The proportion of larvae matching the image shown is indicated in the top right corner of each image. Scale bar applies to all images. C_t_ values of qPCR data are listed in Supplementary Table 1. Source data

To test further whether cardiac fusion requires the DNA-binding or dimerization domain of Hand2, we evaluated whether previously characterized DNA-binding-deficient or dimerization-deficient variants of Hand2 could fully rescue the cardia bifida phenotype in *hand2* mutants. Replacement of three arginines (residues 109–111) within the bHLH domain of mouse HAND2 with the acidic residues aspartic acid and glutamic acid (EDE) has been shown to abolish DNA binding^25,26^. The HAND2 bHLH domain including these three arginine residues is highly conserved (Extended Data Fig. 2a)^27^. In addition, the replacement of a phenylalanine (residue 119) with a proline in mouse HAND2 has been shown to disrupt its dimerization^26^. This highly conserved amino acid is also present in zebrafish Hand2 (Extended Data Fig. 2a)^27^. Previous data obtained from overexpressing these variants in zebrafish embryos indicated that cardiomyocyte production is dependent on the dimerization domain of Hand2 but not on its DNA-binding domain^27^. Therefore, we first integrated these Hand2 variants (Hand2 EDE and Hand2 P; Fig. 2c) into the *hand2 eh22:EGFP* plasmid, injected them into *hand2 FLD*^−/−^ embryos at the one-cell stage and examined cardiac morphology at 48 hpf. Consistent with our mutant zebrafish analysis (Fig. 1d,e), expressing the DNA-binding-deficient form of Hand2 (Hand2 EDE) in *hand2*-expressing cardiac cells rescued the cardia bifida phenotype in *hand2 FLD*^−/−^ embryos (Extended Data Fig. 5a,a′). However, expressing the dimerization-deficient version of Hand2 (Hand2 P) in *hand2*-expressing cells did not rescue the cardia bifida phenotype in *hand2 FLD*^−/−^ embryos (Extended Data Fig. 5a,a′). Previous reports have also shown that phosphorylation of the evolutionarily conserved threonine and serine residues in the first helix of HAND2 (Extended Data Fig. 2a) is important for protein–protein interactions^42,43^. To determine whether Hand2 phosphorylation is required during early heart development, we also integrated this Hand2 variant (Hand2 AA; Fig. 2c) into the *hand2 eh22:EGFP* plasmid, injected it into *hand2 FLD*^−/−^ embryos at the one-cell stage and examined cardiac morphology at 48 hpf. Interestingly, we observed the rescue of the cardia bifida phenotype in most (five of seven) of the injected *hand2 FLD*^−/−^ embryos (Extended Data Fig. 5a,a′). To investigate further the function of these Hand2 variants, we generated stable lines (*Tg(hand2 eh22:hand2 EDE-p2a-EGFP)*^*bns716*^, *Tg(hand2 eh22:hand2 P-p2a-EGFP)*^*bns717*^ and *Tg(hand2 eh22:hand2 AA-p2a-EGFP)*^*bns718*^) and used them to conduct rescue experiments after confirming that they did not cause a phenotype in WT animals (Extended Data Fig. 5b). In line with previous data^25,27^, we found that the dimerization domain of Hand2, but not its DNA-binding domain, was required to rescue early cardiogenesis in *hand2 FLD*^−/−^ embryos (Fig. 2d–f and Extended Data Fig. 5c,c′). We also observed that the phosphorylation domain of Hand2 was important to rescue early cardiogenesis, although not as important as the dimerization domain (Fig. 2g and Extended Data Fig. 5c,c′). To evaluate *hand2* expression levels in the *hand2 eh22*-driven *hand2* variant-overexpressing cells, we sorted EGFP^+^ cells from 20 hpf *Tg(hand2 eh22:EGFP)*, *Tg(hand2 eh22:hand2-p2a-EGFP)*, *Tg(hand2 eh22:hand2 EDE-p2a-EGFP)*, *Tg(hand2 eh22:hand2 P-p2a-EGFP)* and *Tg(hand2 eh22:hand2 AA-p2a-EGFP)* embryos and performed qPCR. We found significant upregulation of *hand2* expression in 20 hpf *hand2* variant-overexpressing cardiac cells compared with *Tg(hand2 eh22:*EGFP*)*^+^ cardiac cells (Extended Data Fig. 5d). We then examined the stability of Hand2 EDE, Hand2 P and Hand2 AA by western blot analysis of exogenously expressed FLAG-tagged proteins in 14 and 4 hpf zebrafish embryos. We found that Hand2 EDE was as stable as the WT; Hand2 P and Hand2 AA were both less stable than the WT; and Hand2 AA was less stable than Hand2 P (Extended Data Fig. 5e,f). We also used AlphaFold2^44^ to model the structure of Hand2, Hand2 Δ27, Hand2 Δ3, Hand2 EDE, Hand2 P and Hand2 AA. We did not observe any obvious differences besides the shortening of the first helix in Hand2 Δ27 (Extended Data Fig. 5g). Altogether, these protein stability and rescue data for Hand2 P (more stable and no rescue) and Hand2 AA (less stable and some rescue) indicate that protein stability, while affected, is not the main reason for the inability of Hand2 P to rescue. In summary, the mutant and rescue data indicate that the dimerization of Hand2, but not its DNA-binding capability, is necessary for early cardiogenesis in zebrafish.

### scRNA-seq analysis of *hand2* reporter-expressing cells in WT and *hand2* mutant embryos

**Fig. 3:**
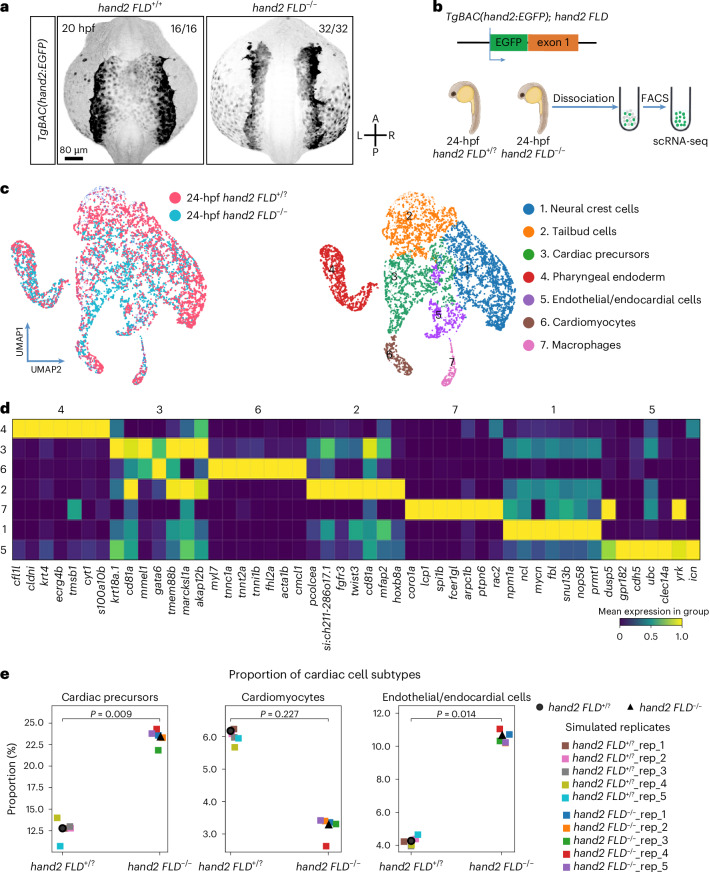
scRNA-seq analysis of *hand2* reporter-expressing cells in WT and *hand2* mutant embryos. **a**, Maximum intensity projections of confocal images of 20 hpf *TgBAC(hand2:*EGFP*)* expression in *hand2 FLD*^+/+^ and *hand2 FLD*^−/−^ sibling embryos. The proportion of embryos matching the image shown is indicated in the top right corner of each image. The scale bar applies to all images. **b**, Schematic of the experimental protocol; the transcriptomes of 3,900 and 3,836 individual *hand2* reporter-expressing cells from 24 hpf *hand2 FLD*^−/−^ and *hand2 FLD*^+/?^ sibling embryos, respectively, were sequenced. **c**, Left, UMAP representation of the cells from *hand2 FLD*^−/−^ (blue) and *hand2 FLD*^+/?^ (pink) sibling embryos. Right, UMAP of the data clustered by the Leiden algorithm. **d**, Heat map of the top 7 DEGs in each cell cluster. A list of the DEGs in each cell cluster can be found in Supplementary Table 2. **e**, Proportional contribution of cardiac precursors, cardiomyocytes and endothelial/endocardial cells in 24 hpf *hand2 FLD*^+/?^ and *hand2 FLD*^−/−^ sibling embryos generated by Scanpro’s bootstrapping method. Panel **b** created with BioRender.com.

**Extended Data Fig. 6:**
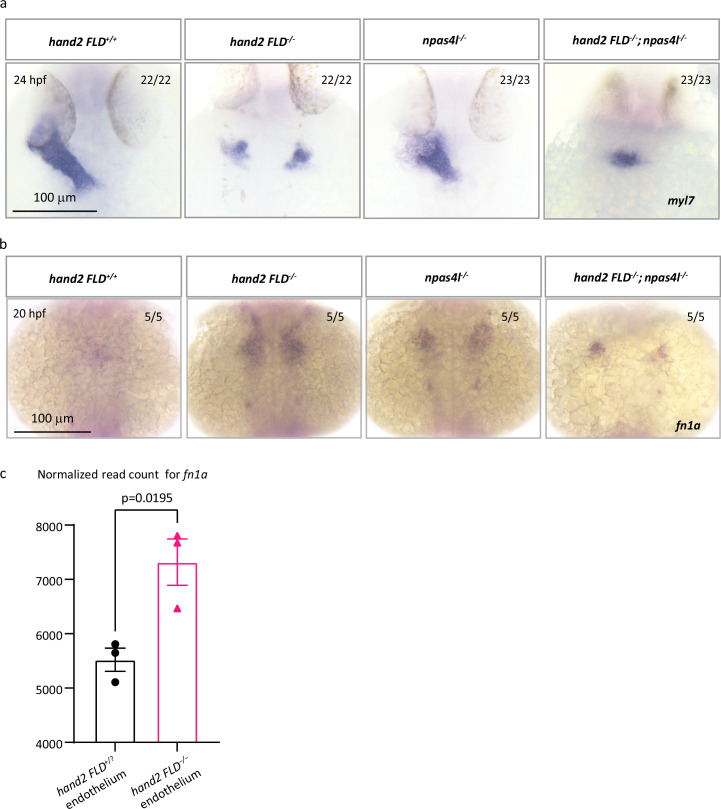
Deleting *cloche/npas4l* promotes myocardial migration in *hand2* mutants. **a**, In situ hybridization showing *myl7* expression in 24 hpf *hand2 FLD*^*+/+*^, *hand2 FLD*^−*/*−^, *npas4l*^−*/*−^, and *hand2 FLD*^−*/*−^; *npas4l*^−*/*−^ sibling embryos. **b**, In situ hybridization showing *fn1a* expression in 20 hpf *hand2 FLD*^*+/+*^, *hand2 FLD*^−*/*−^, *npas4l*^−*/*−^, and *hand2 FLD*^−*/*−^; *npas4l*^−*/*−^ sibling embryos. To prevent overstaining *hand2 FLD*^−*/*−^ and *npas4l*^−*/*−^ embryos, we stopped staining reaction once some embryos became dark. Consequently, *hand2 FLD*^*+/+*^ embryos appear lighter in color. **c**, Normalized read count for *fn1a* expression in *kdrl:*mCherry^*+*^ endothelium from 20 hpf *hand2 FLD*^*+/?*^ and *hand2 FLD*^−*/*−^ sibling embryos. *P* value in c was calculated using an unpaired Student’s *t*-Test. Error bars are mean ± s.e.m.; *n* = 3 biologically independent samples. The proportion of embryos matching the image shown is indicated in the top right corner of each image. Scale bars apply to all images.

Hand2 in zebrafish is necessary for the migration of cardiac precursors; it is also important for the differentiation, patterning and morphogenesis of cardiomyocytes^36^. However, the fate of *hand2* reporter-expressing cells in *hand2 FLD*^−/−^ embryos is unclear; these cells could undergo apoptosis, differentiate into other lineages or arrest in their undifferentiated state. To distinguish between these possibilities, we used the *TgBAC(hand2:EGFP)*^*pd24*^ *hand2* reporter line in combination with the *hand2 FLD* allele to perform in vivo lineage tracing of *hand2*-expressing cells. We observed that *hand2* reporter-expressing cells failed to migrate to the midline in *hand2 FLD*^−/−^ embryos; conversely, in *hand2 FLD*^+/+^ sibling embryos, these cells contributed to the myocardium and endocardium (Supplementary Videos 1 and 2). Subsequent immunostaining for EGFP at 20 hpf confirmed the failure of *hand2* reporter-expressing cardiac cells to migrate to the midline (Fig. 3a). To investigate the underlying molecular changes, we sequenced the transcriptomes of 3,900 and 3,836 individual *hand2* reporter-expressing cells from 24 hpf *hand2 FLD*^−/−^ and *hand2 FLD*^+/?^ sibling embryos, respectively (Fig. 3b). To define and delineate the various clusters, we iteratively fine-tuned the UMAP (uniform manifold approximation and projection) embedding parameters, focusing on the ‘spread’, which defines the dispersion of data points, and ‘minimum distance’, which specifies the closest proximity of any two points in the embedded space. We calibrated the spread within a 1.0–2.0 range and the minimum distance between 0.1 and 1.0; a resolution of seven clusters provided the optimal representation of these transcriptomic data (Fig. 3c). These clusters were subsequently annotated based on the top 7 cluster-specific marker gene expression and prior single-cell RNA-sequencing (scRNA-seq) data from similar embryonic stages (Fig. 3d); a list of the differentially expressed genes (DEGs) in each cell cluster can be found in Supplementary Table 2. To determine whether there were changes in the proportion of the different cell types within *hand2* reporter-expressing cells in *hand2 FLD*^−/−^ and *hand2 FLD*^+/?^ sibling embryos, we used Scanpro’s bootstrapping method^45^ and observed a reduction in *hand2 FLD*^−/−^ embryos in the contribution of *hand2* reporter-expressing cells to the high *myl7*-expressing cardiomyocytes (cluster 6) (Fig. 3e). This cluster comprised <5% of the total number of cells in *hand2 FLD*^−/−^ embryos, which could explain the failure to identify the reduction as significant when sampling it in five simulated replicates. Nevertheless, this observation is in line with previous findings^14,29^. Interestingly, the contribution of *hand2* reporter-expressing cells to the cardiac precursors (cluster 3) and endothelium/endocardium (cluster 5) was significantly expanded in *hand2 FLD*^−/−^ compared with *hand2 FLD*^+/?^ sibling embryos (Fig. 3e). Together, these results indicate that Hand2 has a critical role in driving the differentiation of cardiac precursors into cardiomyocytes. To investigate the potential significance of the increased proportion of the endothelial/endocardial cluster in the *hand2* reporter-expressing cell population in *hand2* mutants, we deleted endothelial/endocardial cells using a *cloche/npas4l* mutation^46–48^. Interestingly, we observed a rescue of the cardia bifida phenotype in *hand2 FLD*^−/−^; *npas4l*^−/−^ embryos (Extended Data Fig. 6a). In addition, we observed that *fn1a* expression was upregulated in the cardiac region (Extended Data Fig. 6b) as well as in endothelial cells (Extended Data Fig. 6c) of 20 hpf *hand2 FLD*^−/−^ embryos. These results are in line with a previous report showing that *fn1a* expression in the anterior LPM region at 17–20 hpf is largely restricted to the endocardial progenitors in WT embryos^49^, as well as with the observed increase in the proportion of the endothelial/endocardial cluster in the *hand2* reporter-expressing cell population in *hand2* mutants (Fig. 3e). Incidentally, this earlier study^49^ also showed the absence of midline *fn1a* expression in 17 hpf *npas4l* mutants, similar to our observation (Extended Data Fig. 6b). We further observed a decrease in *fn1a* mRNA levels in *hand2 FLD*^−/−^; *npas4l*^−/−^ embryos at 20 hpf compared with single-mutant siblings (Extended Data Fig. 6b). Notably, the reduction of *fn1a* expression in *hand2*^−/−^; *nat/fn1a*^+/−^ embryos also rescues the cardia bifida phenotype^37^. Altogether, these data suggest that the increased proportion of the endothelial/endocardial cluster in the *hand2* reporter-expressing cell population in *hand2* mutants leads to an increase in *fn1a* expression, which is responsible, at least in part, for the cardiomyocyte migration defect.

### Hand2 regulates *pdgfra* expression to mediate cardiac fusion

**Fig. 4:**
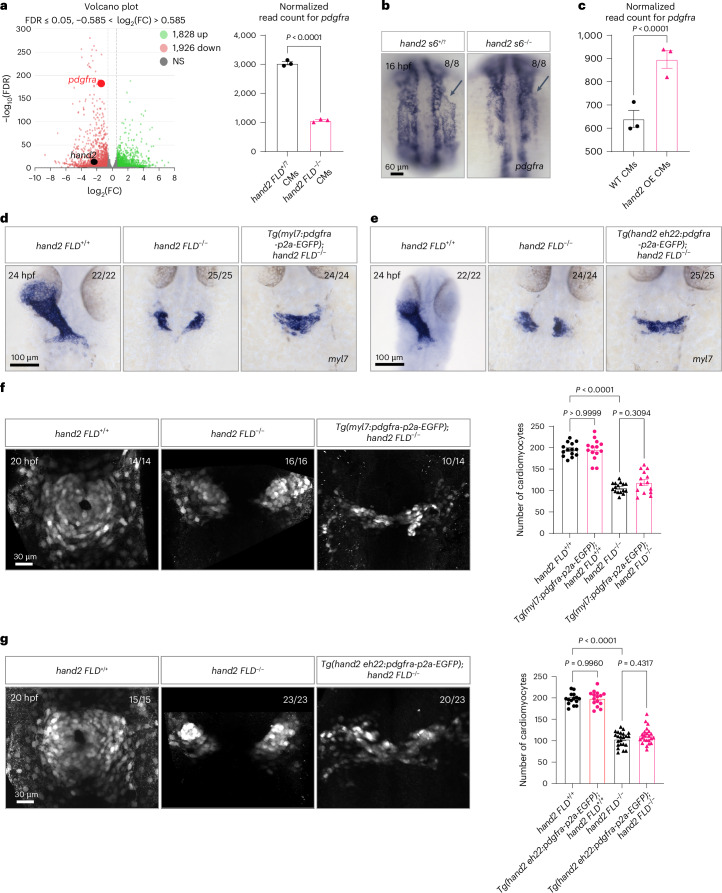
Hand2 regulates *pdgfra* expression to promote cardiac fusion. **a**, Left, transcriptomic analysis was performed using RNA extracted from sorted *Tg(myl7:*EGFP*)*^+^ cells from 20 hpf *hand2 FLD*^+/?^ and *hand2 FLD*^−/−^ sibling embryos. Right, normalized read count for *pdgfra* in 20 hpf *hand2 FLD*^+/?^ and *hand2 FLD*^−/−^ sibling *Tg(myl7:*EGFP*)*^+^ cells; error bars are mean ± s.e.m.; *n* = 3 biologically independent samples. **b**, In situ hybridization showing *pdgfra* expression in the anterior LPM (arrows) of 16 hpf *hand2 s6*^+/?^ and *hand2 s6*^−/−^ sibling embryos. **c**, Normalized read count for *pdgfra* in 20 hpf *hand2* OE and WT sibling *Tg(myl7:*EGFP*)*^+^ cardiomyocytes; error bars are mean ± s.e.m.; *n* = 3 biologically independent samples. **d**, In situ hybridization showing *myl7* expression in 24 hpf *hand2 FLD*^+/+^, *hand2 FLD*^−/−^ and *Tg(myl7:pdgfra-p2a-EGFP)*; *hand2 FLD*^−/−^ sibling embryos. **e**, In situ hybridization showing *myl7* expression in 24 hpf *hand2 FLD*^+/+^, *hand2 FLD*^−/−^ and *Tg(hand2 eh22:pdgfra-p2a-EGFP)*; *hand2 FLD*^−/−^ sibling embryos. **f**, Left, *Tg(myl7:*EGFP*)*^+^ cells in 20 hpf *hand2 FLD*^+/+^, *hand2 FLD*^−/−^ and *Tg(myl7:pdgfra-p2a-EGFP)*; *hand2 FLD*^−/−^ sibling embryos (of the 14 *Tg(myl7:pdgfra-p2a-EGFP)*; *hand2 FLD*^−/−^ embryos, 10 displayed a comparable number of *myl7:*EGFP^+^ cells as *hand2 FLD*^−/−^ embryos and 4 displayed a few more *myl7:*EGFP^+^ cells). Right, quantification of cardiomyocyte numbers in 20 hpf *hand2 FLD*^+/+^ (*n* = 14), *hand2 FLD*^−/−^ (*n* = 16), *Tg(myl7:pdgfra-p2a-EGFP)*; *hand2 FLD*^+/+^ (*n* = 16) and *Tg(myl7:pdgfra-p2a-EGFP)*; *hand2 FLD*^−/−^ (*n* = 14) sibling embryos; error bars are mean ± s.e.m. **g**, Left, *Tg(myl7:*EGFP*)*^+^ cells in 20 hpf *hand2 FLD*^+/+^, *hand2 FLD*^−/−^ and *Tg(hand2 eh22:pdgfra-p2a-EGFP)*; *hand2 FLD*^−/−^ sibling embryos (of the 23 *Tg(hand2 eh22:pdgfra-p2a-EGFP)*; *hand2 FLD*^−/−^ embryos, 20 displayed a comparable number of *myl7:*EGFP^+^ cells as *hand2 FLD*^−/−^ embryos and 3 displayed a few more *myl7:*EGFP^+^ cells). Right, quantification of cardiomyocyte numbers in 20 hpf *hand2 FLD*^+/+^ (*n* = 15), *hand2 FLD*^−/−^ (*n* = 23), *Tg(hand2 eh22:pdgfra-p2a-EGFP)*; *hand2 FLD*^+/+^ (*n* = 23) and *Tg(hand2 eh22:pdgfra-p2a-EGFP)*; *hand2 FLD*^−/−^ (*n* = 23) sibling embryos; error bars are mean ± s.e.m. *P* values were calculated using an unpaired Student’s *t* test (**a** (right), **c**) or a one-way analysis of variance (ANOVA) multiple-comparison test (**f** (right), **g** (right)). All embryos are shown in dorsal views, anterior to the top. The proportion of embryos matching the image shown is indicated in the top right corner of each image. The scale bars apply to all images. FDR, false discovery rate; FC, fold change; CMs, cardiomyocytes; NS, not significant (*P* > 0.05).

**Extended Data Fig. 7:**
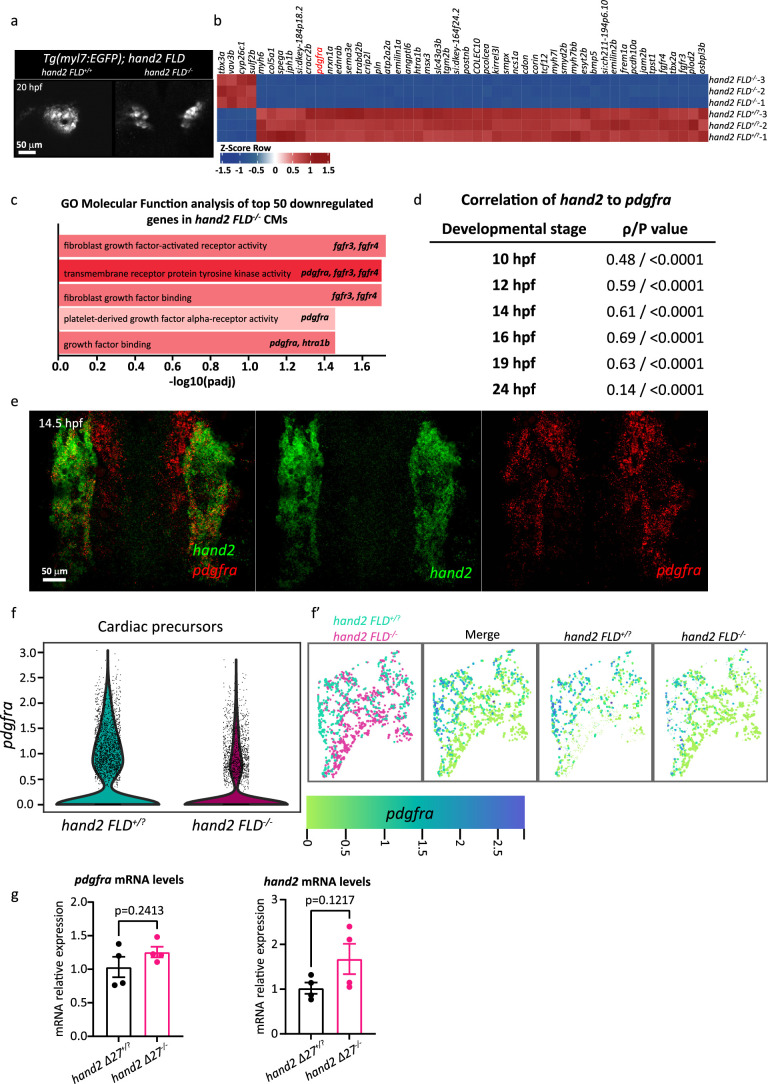
Hand2 promotes *pdgfra* expression in zebrafish cardiomyocytes. **a**, Maximum intensity projections of confocal images of *Tg(myl7:*EGFP*)* expression in 20 hpf *hand2 FLD*^*+/+*^ and *hand2 FLD*^−*/*−^ sibling embryos. **b**, Heat map of DEGs comparing the *Tg(myl7:*EGFP*)* ^*+*^ cells *hand2 FLD*^*+/?*^ and *hand2 FLD*^−*/*−^ sibling embryos at 20 hpf. **c**, Gene ontology (GO) term analysis of molecular function shows enrichment of platelet-derived growth factor alpha-receptor activity genes downregulated in 20 hpf *hand2 FLD*^−*/*−^ *Tg(myl7:*EGFP*)*^*+*^ cells. **d**, Spearman correlation coefficient (ρ) between *hand2* expression and *pdgfra* expression during early embryonic stages from the Zebrahub datasets. **e**, Double fluorescence *in situ* hybridization showing *hand2* (green) and *pdgfra* (red) expression patterns illustrating their overlap in the 14.5 hpf anterior LPM. **f**, Violin plots showing *pdgfra* expression within cluster 3 (cardiac precursors). f’ UMAP representation of cells from different samples within cluster 3. **g**. Relative mRNA levels of *pdgfra* and *hand2* in *Tg(myl7*:EGFP)^+^ cells from 24 hpf *hand2 Δ27*^*+/?*^ and *hand2 Δ27*^−*/*−^ sibling embryos; error bars are mean ± s.e.m.; *n* = 4 biologically independent samples. *P* values in g were calculated using an unpaired Student’s *t*-Test. Scale bars apply to all images.

**Extended Data Fig. 8:**
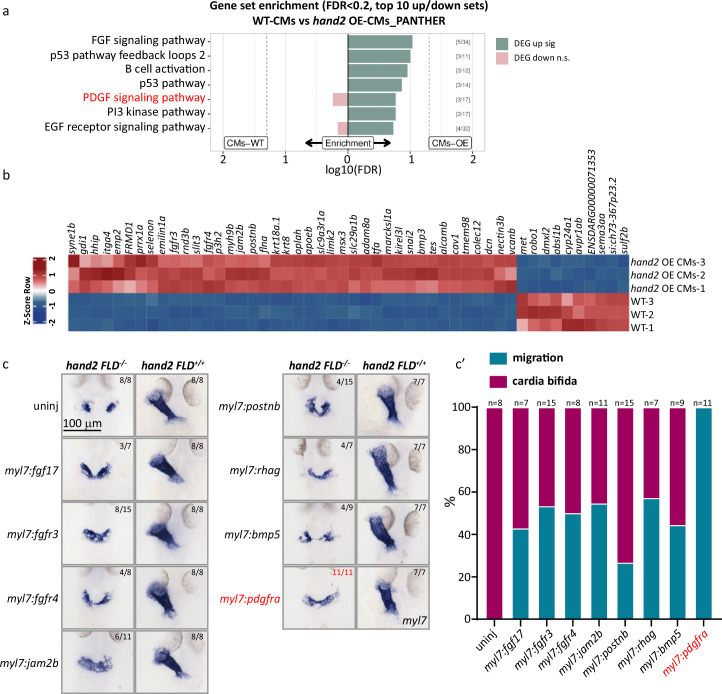
*pdgfra* is a potential effector of Hand2 during early zebrafish cardiogenesis. **a**, Gene set enrichment analysis (GSEA) revealed that the PDGF signaling pathway is enriched in *hand2* OE myocardial cells. **b**, Heat map of DEGs comparing the *Tg(myl7:*mCherry*)*^*+*^ cells in *Tg(myl7:hand2-p2a-EGFP)* and WT sibling embryos. **c**, In situ hybridization showing *myl7* expression in 24 hpf *hand2 FLD*^*+/+*^ and *hand2 FLD*^−*/*−^ sibling embryos that were injected at the one-cell stage with a *myl7:fgf17*, *myl7:fgfr3*, *myl7:fgfr4*, *myl7:jam2b*, *myl7:postnb*, *myl7:rhag*, *myl7:bmp5*, or *myl7:pdgfra* plasmid. c’, Percentage of 24 hpf *hand2 FLD*^−*/*−^ embryos that displayed evidence of cardiac cell migration after injection at the one-cell stage with a *myl7:fgf17*, *myl7:fgfr3*, *myl7:fgfr4*, *myl7:jam2b*, *myl7:postnb*, *myl7:rhag*, *myl7:bmp5*, or *myl7:pdgfra* plasmid. All embryos are shown in dorsal views, anterior to the top. The proportion of embryos matching the image shown is indicated in the top right corner of each image. Scale bar applies to all images.

To gain mechanistic insight into how Hand2 regulates cardiac fusion, we performed transcriptomic analysis of myocardial cells from *hand2 FLD*^−/−^ and *hand2 FLD*^+/?^ sibling embryos at 20 hpf (Fig. 4a), a stage when the cardiomyocyte migration defect first becomes apparent (Extended Data Fig. 7a). As Hand2 acts primarily as a transcriptional activator^50^, we prioritized the genes downregulated in *hand2 FLD*^−/−^ cardiomyocytes compared with *hand2 FLD*^+/?^ sibling cardiomyocytes (Extended Data Fig. 7b). For the gene ontology analysis, we focused on the top 50 downregulated genes (Extended Data Fig. 7c) and identified *pdgfra* as having significantly decreased read count in *hand2 FLD*^−/−^ cardiomyocytes compared with *hand2 FLD*^+/?^ sibling cardiomyocytes (Fig. 4a, right). Platelet-derived growth factor receptor-α (PDGFRα) is a tyrosine kinase receptor conserved in vertebrates. Previous studies have shown that PDGFRα is important for the migration of a number of cell types, including cardiac cells, in mice^51^ and zebrafish^51,52^. Interestingly, in zebrafish, *pdgfra* expression in the anterior LPM colocalizes with *hand2* expression^51^. However, the relationship between *hand2* and *pdgfra* remains poorly understood. To investigate this relationship further, we first examined the coexpression of *hand2* and *pdgfra* using a large zebrafish scRNA-seq dataset from Zebrahub^53^, covering a developmental time course from the end of gastrulation to 10 dpf. By analyzing the mesoderm and LPM cells at 10, 12, 14, 16, 19 and 24 hpf, we observed a significant correlation between *hand2* and *pdgfra* expression, with their coexpression increasing from 12 to 16 hpf (Extended Data Fig. 7d). This observation is consistent with previous studies showing that *pdgfra* is expressed in the anterior LPM, where *hand2* is also expressed (Extended Data Fig. 7e)^51^. Consistent with these data, we found that *pdgfra* was mainly expressed in the cardiac precursors (cluster 3) within the *hand2* reporter-expressing scRNA-seq dataset of WT embryos but not in *hand2 FLD*^−/−^ embryos (Extended Data Fig. 7f,f′). We also analyzed *pdgfra* mRNA levels in *hand2* DNA-binding and phosphorylation domain mutants by qPCR (Extended Data Fig. 7g) and found no significant differences compared with WT siblings. To investigate this observation further, we performed in situ hybridization for *pdgfra* expression and observed its downregulation in the anterior LPM region of *hand2 s6*^−/−^ embryos compared with their WT siblings at 16 hpf (Fig. 4b). To test further whether Hand2 positively regulates *pdgfra* in cardiomyocytes, we performed transcriptomic analyses of cardiomyocytes from 20 hpf *hand2*-overexpressing (*hand2* OE; that is, *Tg(myl7:hand2-p2a-EGFP)*) and WT sibling embryos and found significantly upregulated *pdgfra* expression (Fig. 4c). Moreover, gene ontology analysis revealed that the PDGF signaling pathway was enriched upon overexpression of *hand2* in the myocardium (Extended Data Fig. 8a). Altogether, these results indicate that Hand2 positively regulates *pdgfra* expression levels in zebrafish cardiomyocytes.

To identify functional effectors of Hand2, the full coding sequences of the eight genes that were most downregulated in *hand2 FLD*^−/−^ cardiomyocytes (Extended Data Fig. 7b) and upregulated in *hand2* OE cardiomyocytes (Extended Data Fig. 8b) were integrated into plasmids behind a *myl7* promoter. We injected these plasmids into *hand2 FLD*^−/−^ embryos at the one-cell stage and examined cardiomyocyte migration. We found that myocardial expression of *pdgfra* uniquely led to a consistent rescue of myocardial migration in all injected *hand2 FLD*^−/−^ embryos (Extended Data Fig. 8c,c′). To investigate further the relationship between Hand2 function and *pdgfra* expression, we generated the *Tg(hand2 eh22:pdgfra-p2a-EGFP)*^*bns666*^ and *Tg(myl7:pdgfra-p2a-EGFP)*^*bns662*^ lines to assess whether overexpression of *pdgfra* in cardiac precursors and/or myocardial cells could rescue the *hand2 FLD*^−/−^ phenotype. Notably, we observed a rescue of myocardial migration in *hand2 FLD*^−/−^ embryos with both lines (Fig. 4d–g). Furthermore, compared with controls, the number of *myl7*:EGFP^+^ cells did not appear to be significantly different in *Tg(myl7:pdgfra-p2a-EGFP)*; *hand2 FLD*^−/−^ embryos (4 of 14 contained a few more *myl7:*EGFP^+^ cells) (Fig. 4f) or *Tg(hand2 eh22:pdgfra-p2a-EGFP)*; *hand2 FLD*^−/−^ embryos (3 of 23 contained a few more *myl7:*EGFP^+^ cells) (Fig. 4g). Taken together, these results indicate that Hand2 positively regulates *pdgfra* expression to promote cardiac fusion in zebrafish.

### Identification of putative Hand2-binding proteins

**Fig. 5:**
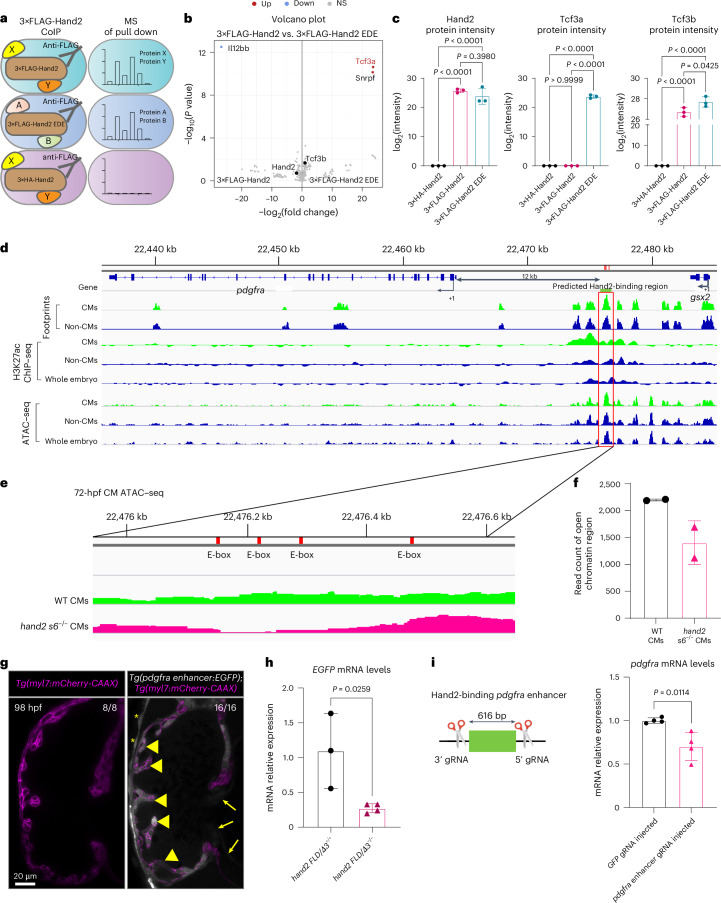
Deletion of a putative Hand2-binding region in a zebrafish *pdgfra* enhancer results in decreased *pdgfra* expression in the embryonic heart. **a**, Illustration of the CoIP–MS experiment: 14 hpf *3×FLAG-hand2* and *3×FLAG-hand2 EDE* mRNA-injected embryos were subjected to FLAG CoIP reactions, controlling for background with *3×HA-hand2* mRNA-injected embryos. The pulled complexes were subjected to MS. **b**, Volcano plot showing significantly enriched proteins in the FLAG CoIP–MS experiment. Significant proteins are indicated as blue (down) and red (up) dots, and nonsignificant proteins are indicated in gray. **c**, Protein intensity of Hand2, Tcf3a and Tcf3b in 3×HA-Hand2, 3×FLAG-Hand2 and 3×FLAG-Hand2 EDE protein complexes by MS; error bars are mean ± s.d.; *n* = 3 biologically independent samples. **d**, Genome browser view showing ATAC–seq and ChIP–seq peaks enriched in myocardial cells at the *pdgfra* locus. Red box: predicted Hand2-binding region. **e**, ATAC–seq genome tracks showing open chromatin regions at the predicted Hand2-binding region in 72 hpf WT (green) and *hand2 s6*^−/−^ (magenta) cardiomyocytes. The tracks show the peak signal intensity for open chromatin regions (data analyzed from GSE120238 (ref. ^55^)). Red boxes: conserved E-boxes in the –12-kb *pdgfra* enhancer. **f**, Normalized read count for the open chromatin region across the predicted Hand2-binding region (from chr20: 22,476,180–22,476,620 bp) in 72 hpf WT and *hand2 s6*^−/−^ cardiomyocytes (data analyzed from GSE120238 (ref. ^55^)); error bars are mean ± s.d.; *n* = 2 biologically independent samples. **g**, Confocal images of hearts from representative 98 hpf *Tg(myl7:*mCherry-CAAX*)* larvae not carrying (left) or carrying (right) the *pdgfra enhancer:EGFP* transgene; EGFP fluorescence is detectable in the heart of the representative double-transgenic larva (in cardiomyocytes (short arrows) and endocardial cells (long arrows) and in some pericardial cells (asterisks)); EGFP is shown in white, and cardiomyocyte membranes are shown in magenta (*myl7:*mCherry-CAAX). **h**, Relative mRNA levels of *EGFP* in 18 hpf *Tg(pdgfra enhancer:EGFP)*; *hand2 FLD/Δ3*^+/+^ and *Tg(pdgfra enhancer:EGFP)*; *hand2 FLD/Δ3*^−/−^ sibling embryos; error bars are mean ± s.d.; *n* = 3 *hand2 FLD/Δ3*^+/+^ and *n* = 4 *hand2 FLD/Δ3*^−/−^. **i**, Left, schematic of the strategy to generate Hand2-binding *pdgfra* enhancer crispant embryos. Right, relative mRNA levels of *pdgfra* in the hearts of *GFP* crispant and Hand2-binding *pdgfra* enhancer crispant embryos at 24 hpf. *P* values were calculated using a one-way ANOVA multiple-comparison test (**c**) or an unpaired Student’s *t* test (**h**, **i** (right)); error bars are mean ± s.d.; *n* = 4 biologically independent samples. The proportion of larvae matching the image shown is indicated in the top right corner of each image. The scale bar applies to all images. *C*_t_ values of qPCR data are listed in Supplementary Table 1.

**Extended Data Fig. 9:**
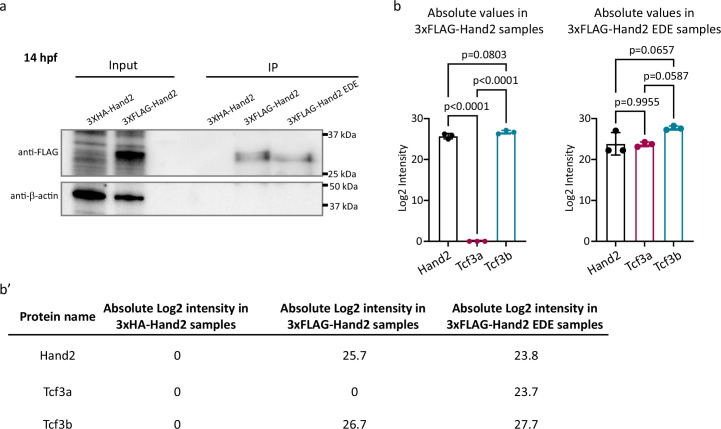
3xFLAG-Hand2 pull down experiment. **a**, FLAG IP of lysates from 14 hpf *3xFLAG-hand2* and *3xFLAG-hand2 EDE* mRNA injected embryos blotted with antibodies against FLAG and β-actin. **b**, b’, Absolute protein intensity of Hand2, Tcf3a, and Tcf3b in 3xFLAG-Hand2 and 3xFLAG-Hand2 EDE protein complexes by mass spectrometry; error bars are mean ± s.d.; *n* = 3 biologically independent samples; list of the mean of absolute protein intensity shown in b’.

To gain insight into how Hand2 regulates *pdgfra* expression in zebrafish embryos, including without its DNA-binding domain, we performed an unbiased and quantitative coimmunoprecipitation coupled to mass spectrometry (CoIP–MS) experiment to identify Hand2-binding proteins. We injected mRNA encoding 3×FLAG-Hand2 and 3×FLAG-Hand2 EDE into zebrafish embryos at the one-cell stage and collected them at 14 hpf (Fig. 5a and Extended Data Fig. 9a). Intriguingly, we found that transcription factor 3a (Tcf3a) was enriched only in the samples prepared from the *3×FLAG-hand2 EDE* mRNA-injected embryos, whereas it was absent in the samples prepared from the *3×FLAG-hand2* mRNA-injected embryos (Fig. 5b,c and Extended Data Fig. 9b,b′); a list of the interacting proteins in each relevant sample can be found in Supplementary Table 3. Tcf3b was also significantly enriched in the *3×FLAG-hand2 EDE* samples compared with the *3×FLAG-hand2* samples (Fig. 5c). We found that Hand2 and Tcf3b protein abundance was comparable in the *3×FLAG-hand2* and *3×FLAG-hand2 EDE* samples (Fig. 5c and Extended Data Fig. 9b,b′), suggesting that Hand2 homodimers have a role during cardiogenesis. Previous studies in mice have shown that HAND2 binds to TCF3 (also known as E12/E47 and E2a) to regulate its targets^26,33,50,54^. Together, these results suggest that, in zebrafish, the DNA-binding-deficient variant of Hand2 binds more strongly to Tcf3a and Tcf3b to promote the expression of *pdgfra* in cardiac cells.

### Deletion of a putative Hand2-binding region in a zebrafish *pdgfra* enhancer results in decreased *pdgfra* expression in the embryonic heart

To investigate potential coregulatory interactions that promote *pdgfra* expression, we again used the ATAC–seq and H3K27ac ChIP–seq data introduced earlier (Extended Data Fig. 4a,b). We found a putative Hand2/Tcf3-binding site (E-box) in the open chromatin domain upstream of the *pdgfra* transcription start site (Fig. 5d). To test whether this enhancer was responsive to Hand2 binding, we first examined chromatin accessibility in 72 hpf WT and *hand2 s6*^−/−^ cardiomyocytes using a published ATAC–seq dataset^55^. Notably, we observed a reduction in chromatin accessibility in *hand2 s6*^−/−^ cardiomyocytes compared with WT cardiomyocytes (Fig. 5e,f). We then cloned this enhancer element (616 bp) to generate a stable line (*Tg(pdgfra enhancer:EGFP)*^*bns741*^) and observed EGFP expression in the heart, especially in cardiomyocytes (Fig. 5g). To assess whether this *pdgfra* enhancer activity was dependent on Hand2 in vivo, we crossed this *Tg(pdgfra enhancer:EGFP)* reporter line into the *hand2* mutant background. qPCR analysis showed a significant reduction of *EGFP* mRNA levels in 18 hpf *Tg(pdgfra enhancer:EGFP)*; *hand2 FLD/Δ3*^−/−^ embryos compared with *Tg(pdgfra enhancer:EGFP)*; *hand2 FLD/Δ3*^+/+^ sibling embryos (Fig. 5h). To investigate the function of this enhancer further, we designed and validated gRNAs around this region and injected them to delete the enhancer region mosaically (Fig. 5i, left). We observed a significant reduction in *pdgfra* mRNA levels in the hearts of gRNA-injected embryos compared with *GFP* gRNA-injected controls at 24 hpf (Fig. 5i, right). Altogether, these data suggest that the activity of this zebrafish *pdgfra* enhancer in cardiac cells is modulated by Hand2, possibly through direct binding.

### HAND2 promotes *Pdgfra* expression in mouse cardiac cells

**Fig. 6:**
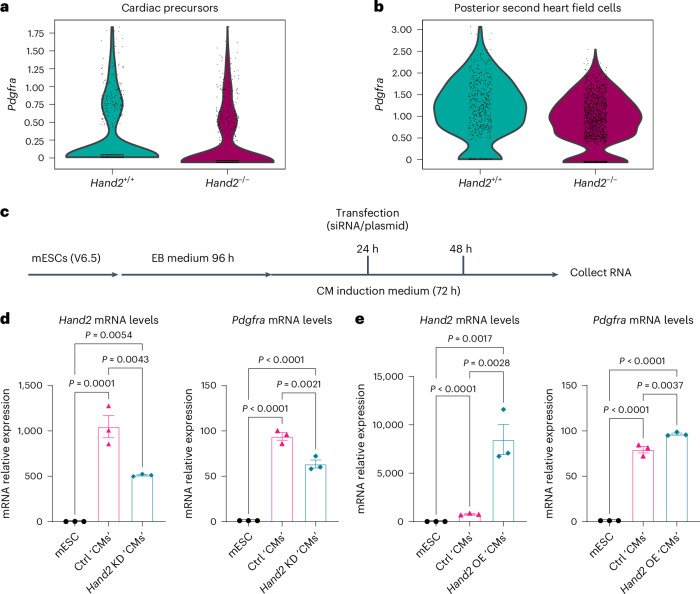
*Hand2* promotes *Pdgfra* expression in mouse cardiac cells. **a**, Violin plots showing *Pdgfra* expression in cardiac precursors of E8.25 WT and *Hand2*^−/−^ mutant hearts (data analyzed from GSE126128 (ref. ^56^)). **b**, Violin plots showing *Pdgfra* expression in posterior second heart field cells of E8.25 WT and *Hand2*^−/−^ mutant hearts (data analyzed from GSE126128 (ref. ^56^)). **c**, Schematic of knockdown and overexpression of *Hand2* during mESC differentiation into cardiac cells. **d**, Relative mRNA levels of *Hand2* and *Pdgfra* in *Hand2* knockdown mESC-derived cardiac cells; error bars are mean ± s.e.m.; *n* = 3 biologically independent samples. **e**, Relative mRNA levels of *Hand2* and *Pdgfra* in *Hand2* OE mESC-derived cardiac cells; error bars are mean ± s.e.m.; *n* = 3 biologically independent samples. *P* values in **d** and **e** were calculated using a one-way ANOVA multiple-comparison test. The average mRNA level in mESCs was set at 1.0. The *C*_t_ values of qPCR data are listed in Supplementary Table 1. Ctrl, control; KD, knockdown.

**Extended Data Fig. 10:**
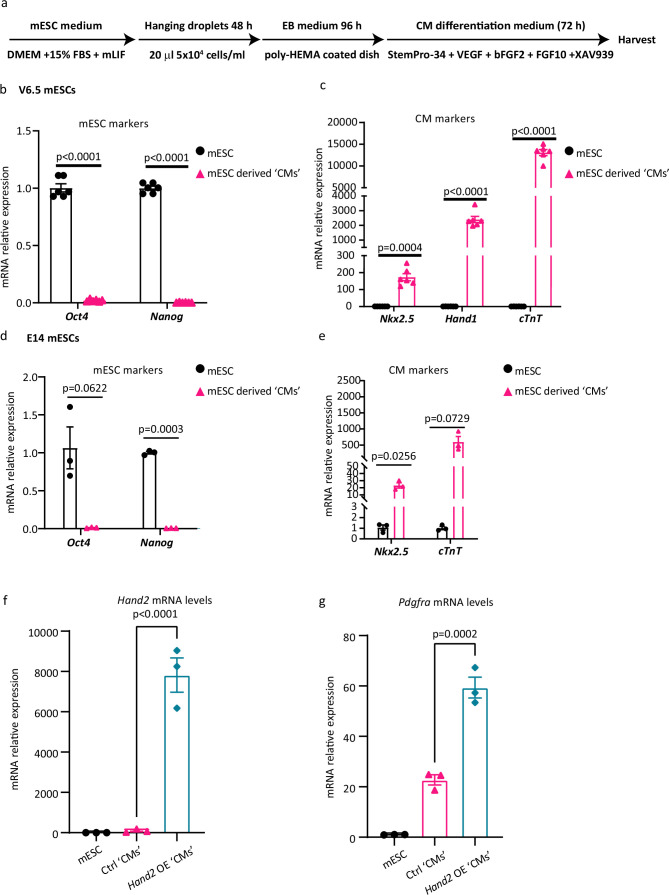
mESC differentiation into cardiac cells. **a**, Schematic of mESC differentiation into cardiac cells. **b**, Relative mRNA levels of mESC (pluripotency) markers in mESC (V6.5)-derived cardiac cells; error bars are mean ± s.e.m.; *n* = 6 biologically independent samples. **c**, Relative mRNA levels of CM markers in mESC (V6.5)-derived cardiac cells; error bars are mean ± s.e.m.; *n* = 6 biologically independent samples. **d**, Relative mRNA levels of mESC (pluripotency) markers in mESC (E14)-derived cardiac cells; error bars are mean ± s.e.m.; *n* = 3 biologically independent samples. **e**, Relative mRNA levels of CM markers in mESC (E14)-derived cardiac cells; error bars are mean ± s.e.m.; *n* = 3 biologically independent samples. **f**, Relative mRNA levels of *Hand2* in *Hand2* OE mESC (E14)-derived cardiac cells; error bars are mean ± s.e.m.; *n* = 3 biologically independent samples. **g**, Relative mRNA levels of *Pdgfra* in *Hand2* OE mESC (E14)-derived cardiac cells; error bars are mean ± s.e.m.; *n* = 3 biologically independent samples. *P* values were calculated using an unpaired Student’s *t*-Test in b-e, and using a one-way ANOVA multiple comparison test in f, g. The average mRNA level in mESCs was set at 1.0. C_t_ values of qPCR data are listed in Supplementary Table 1.

To explore the relevance of the HAND2–*Pdgfra* axis in mammals, we first examined *Pdgfra* expression in mice using a published scRNA-seq dataset from E8.25 WT and *Hand2*^−/−^ hearts^56^. Notably, we observed a reduction in *Pdgfra* expression in E8.25 *Hand2*^−/−^ cardiac precursors and posterior second heart field cells (Fig. 6a,b). We also analyzed a published HAND2 ChIP–seq dataset from E10.25–E10.5 mouse hearts^57^ and observed a significant occupancy peak in the *Pdgfra* locus (Supplementary Fig. 1), further reinforcing the link between HAND2 function and *Pdgfra* expression in embryonic mouse hearts. We then used mESC-derived cardiac cells^58^ to knock down and overexpress *Hand2* in cardiac cells (Fig. 6c and Extended Data Fig. 10a). Beating mESC-derived ‘cardiomyocytes’ were visible within 30–48 h of treatment with cardiomyocyte induction medium (Supplementary Video 3), with significant downregulation of pluripotency genes (*Oct4* and *Nanog*) (Extended Data Fig. 10b) and upregulation of cardiomyocyte genes (*Nkx2.5*, *Hand1* and *cTnT*) (Extended Data Fig. 10c). Notably, knockdown of *Hand2* expression in mESC-derived cardiac cells led to decreased *Pdgfra* mRNA levels (Fig. 6d). In contrast, *Hand2* overexpression in mESC-derived cardiac cells led to increased *Pdgfra* mRNA levels (Fig. 6e). To exclude the possibility that the increase in *Pdgfra* mRNA levels caused by *Hand2* overexpression was mESC line dependent, we repeated the experiments using another mESC line (E14 ESC line) and obtained comparable results (Extended Data Fig. 10d–g).

Altogether, these results indicate that, in mice as in zebrafish, HAND2 positively regulates *Pdgfra* expression in cardiac cells.

## Discussion

Our loss- and gain-of-function analyses in zebrafish showed that the function of Hand2 is dependent on its dimerization domain but not its DNA-binding and phosphorylation domains, particularly in the context of pectoral fin formation and early cardiogenesis. To gain a better understanding of Hand2 function in early cardiogenesis, we performed transcriptomic analyses and a phenotypic rescue assay and identified *pdgfra* as a Hand2 target and effector. Furthermore, mosaic deletion of a putative Hand2/Tcf3-responsive *pdgfra* enhancer resulted in decreased *pdgfra* expression in early zebrafish hearts. In addition, HAND2 also appeared to promote *Pdgfra* expression in embryonic mouse hearts and mESC-derived cardiac cells. Collectively, these results indicate that HAND2 regulates early cardiogenesis through its dimerization domain and by promoting *Pdgfra* expression.

Previous genetic studies have shown that HAND2 can function independently of direct DNA binding in the context of early cardiogenesis in mice^25,50^. Furthermore, overexpression of *hand2* in zebrafish embryos can enhance cardiomyocyte production, and the dimerization domain of Hand2 but not its DNA-binding domain is required for this activity^27^. In this study, we generated precise mutations in zebrafish *hand2* and found that the heart formed normally in *hand2* DNA-binding and phosphorylation domains-deficient (*hand2 Δ27*^−/−^) embryos, although with a noticeable delay (Fig. 1). However, *hand2 Δ27*^−/−^ animals do not survive to the juvenile stage, indicating that Hand2 acts in both a DNA-binding-independent and -dependent manner. These findings are consistent with genetic studies in mice showing that the *Hand2* DNA-binding-deficient (*Hand2*^EDE/EDE^) mutants die 1–3 days later than the global *Hand2*^−/−^ mutants^25^.

We generated a *hand2* FLD allele to help determine the null phenotype. *hand2* dimerization-deficient (*hand2 Δ3*^−/−^) embryos also display the null phenotype, including cardiogenesis and pectoral fin formation defects, demonstrating that dimerization of Hand2 is a critical determinant of Hand2 function. The rescue data with the Hand2 variants indicated that the phosphorylation domain of Hand2 is important for its function, whereas the mutant analysis (that is, *hand2 Δ27*) indicated otherwise. A previous study^42^ has shown that changes in the phosphorylation of mouse HAND1 modulate its function by regulating its ability to form HAND1–HAND1 homodimers versus HAND1 heterodimers. Based on these data, we hypothesized that the zebrafish Hand2 AA (phosphorylation-deficient) variant tends to form homodimers rather than heterodimers. Mutating the phosphorylation domain in the endogenous *hand2* locus will allow one to test its function further. These observations are in line with previous studies showing that HAND2 forms homodimers, or heterodimers with ubiquitous bHLH proteins (E proteins), to assemble a bipartite DNA-binding domain that enables recognition of the E-box consensus sequence (CANNTG), thereby resulting in transcriptional activation or repression^50,59^. Our CoIP–MS results indicated that differences in the interaction between the E proteins Tcf3a/Tcf3b and the DNA-binding-deficient variant of Hand2, compared with WT Hand2, explain their different effects on cardiac development. Notably, there is only 53.13% similarity between zebrafish Tcf3a and Tcf3b, although there is 93.90% similarity between their bHLH domains^60^, which is involved in DNA binding and protein interaction^61^ (Supplementary Fig. 2). Altogether, these results suggest that the DNA-binding-deficient variant of Hand2 (Hand2 EDE) binds to Tcf3a (and Tcf3b) more strongly than the WT Hand2. Thus, Hand2 homodimers (and possibly Hand2 EDE homodimers) as well as Hand2/Tcf3b and Hand2 EDE/Tcf3a/Tcf3b heterodimers would recognize the E-boxes involved in regulating Hand2 targets. We propose that, similar to Hand2 EDE, the Hand2 variant encoded by *hand2 Δ27* also binds to Tcf3a (and Tcf3b) more strongly than the WT Hand2. Additional work will be required to understand why Hand2 EDE binds to Tcf3a (and Tcf3b) more strongly than the WT Hand2. It is, of course, interesting to think about this new interaction of Hand2 EDE in the context of genetic compensation, a topic under renewed interest^62^. In addition, the phenotypic difference between *hand2 Δ3* mutants (cardia bifida) and *hand2 Δ27* mutants (slight delay in cardiac development) could be due to Hand2 ∆27 retaining its ability to dimerize. However, we cannot rule out that Hand2 ∆27 is more stable than Hand2 ∆3, and thus, differences in protein stability might also contribute to the observed differences in phenotypes. In addition, in animals in which *HAND1* is present, HAND2 might form heterodimers with HAND1 to promote cardiac development^15,42,43^. Thus, identifying HAND1/2’s other partners in organisms that also have *HAND1* will further our understanding of their functions.

Previous studies have shown that PDGFRα is important for the movement of a number of cell types, including cranial neural crest cells and myocardial cells in mice^51,63^ and cardiac cells in zebrafish^51,52^. Mouse embryos lacking PDGFRα function also exhibit other cardiac defects, including enlarged hearts, septal defects, reduced myocardial wall thickness and abnormal valves^63,64^. However, the relationship between Hand2 and *pdgfra* has not been clear.

Our transcriptomic analyses, phenotypic rescue data, CoIP–MS results and enhancer investigations in zebrafish indicated that Hand2 promotes *pdgfra* expression during early cardiogenesis. HAND2 has been shown to promote *Pdgfra* expression in the mouse epicardium^65^, and luciferase transactivation data in human embryonic kidney cells in culture suggest direct *PDGFRA* regulation by HAND2 (ref. ^65^). These data indicate that *PDGFRA* is also a target of HAND2 in cells other than cardiomyocytes. In addition, HAND2 ChIP–seq data from E10.25–E10.5 mouse hearts show significant occupancy peaks, which contain putative HAND2-binding sites (E-boxes), in the *Pdgfra* locus (Supplementary Fig. 1)^57^. Altogether, these data indicate that HAND2 in the form of homodimers or heterodimers positively regulates *PDGFRA* expression, thereby promoting cell migration during early cardiogenesis. Given all these data, as well as the many other reported roles of HAND2 and PDGFRA in mammalian development^66,67^, including cardiogenesis^8,51,57,63,67–69^, we anticipate that double heterozygosity for deleterious mutations in *HAND2* and *PDGFRA* may lead to congenital malformations, including cardiac malformations.

## Methods

### Zebrafish husbandry and lines

Zebrafish larvae were reared under standard conditions. Adult fish were maintained in 3.5 l tanks at a stocking density of 10 fish/l with the following parameters: water temperature: 27–27.5 °C; light:dark cycle: 14:10; pH: 7.0–7.5; conductivity: 750–800 µS cm^−1^. The fish were fed with granulated and live food (Artemia salina) 3 to 5 times per day, depending on their age. Health was monitored twice a year. All procedures performed on animals were conducted in accordance with the guidelines of the European Parliament Directive 2010/63/EU on the protection of animals used for scientific purposes and have been approved by the Animal Protection Committee (Tierschutzkommission) of the Regierungspräsidium Darmstadt (reference: B2/1218 and B2/9000).

### Zebrafish lines

The following transgenic lines were generated for this study: *Tg(hand2 eh22:EGFP)*^*bns623*^, *Tg(myl7:hand2-p2a-GFP)*^*bns610*^, *Tg(hand2 eh22:hand2-p2a-GFP)*^*bns665*^, *Tg(myl7:pdgfra-p2a-GFP)*^*bns662*^, *Tg(hand2 eh22:pdgfra-p2a-GFP)*^*bns666*^, *Tg(hand2 eh22:hand2 EDE-p2a-EGFP)*^*bns716*^, *Tg(hand2 eh22:hand2 P-p2a-EGFP)*^*bns717*^, *Tg(hand2 eh22:hand2 AA-p2a-EGFP)*^*bns718*^ and *Tg(pdgfra enhancer:EGFP)*^*bns741*^. The following mutant lines were generated for this study: *hand2 FLD*^*bns539*^, *hand2 Δ27*^*bns540*^ and *hand2 Δ3*^*bns603*^.

The following transgenic and mutant lines were also used in this study: *TgBAC(hand2:GFP)*^*pd24*^ (ref. ^70^), *Tg(myl7:EGFP)*^*twu26*^ (ref. ^71^), *Tg(myl7:mCherry-CAAX)*^*bns7*^ (ref. ^72^), *Tg(kdrl:Hsa.HRAS-mCherry)*^*s896*^ (ref. ^73^), *hand2*^*s6*^ (ref. ^14^) and *npas4l Pt(npas4l-p2A-Gal4-VP16)*^*bns313*^ (ref. ^47^).

### Generation of *hand2* mutants using CRISPR–Cas9 technology

To generate the *hand2* mutant lines, we designed gRNAs using a CRISPR design tool (https://www.crisprscan.org/). The gRNAs were assembled as previously described^74,75^ and transcribed using a MEGAshortscript T7 Transcription Kit (Thermo Fisher Scientific). *cas9* mRNA was transcribed using an mMESSAGE mMACHINE T3 Transcription Kit (Thermo Fisher Scientific) with pT3TS-nCas9n as a template. gRNAs and *cas9* RNAs were purified with an RNA Clean and Concentrator Kit (Zymo Research). gRNAs (~20 pg per embryo for each gRNA) and *cas9* mRNA (~30 pg per embryo) were coinjected at the one-cell stage.

To generate the *hand2 FLD*^*bns539*^ mutant line, we used gRNA#1 targeting the 5′ untranslated region (UTR) (CATTGATTCCACAACGTGCT) and gRNA#2 targeting the 3′ UTR (TGTGTGTTGCTGTCTGATAT), resulting in the isolation of a 1,411-bp-deletion allele.

To generate the *hand2 Δ27*^*bns540*^ mutant line, we used gRNA#3 (AAAGGAGAGGCGCAGGACTC), resulting in the isolation of a 27-bp-deletion allele.

To generate the *hand2 Δ3*^*bns603*^ mutant line, we used gRNA#4 (TTGCAGAACTCAGGGAATGC), resulting in the isolation of a 3-bp-deletion allele.

To mosaically delete the *pdgfra* enhancer, we used gRNA#1 (GGCGGAGGAGCGCAGCACAA), gRNA#2 (GGGGCCGTGGAGCAGGACGG) and gRNA#3 (GGGGAATGCACAATTACATT).

### Generation of transgenic lines

To generate the *Tg(hand2 eh22:EGFP)*^*bns623*^ line, we cloned a 752-bp *hand2* enhancer (*eh22*) sequence into a Tol2 backbone.

To generate the *Tg(myl7:hand2-p2a-EGFP)*^*bns610*^ line, we cloned the *hand2-p2a-EGFP* sequences into a Tol2 backbone downstream of 800 bp of the *myl7* promoter.

To generate the *Tg(hand2 eh22:hand2-p2a-EGFP)*^*bns665*^, *Tg(hand2 eh22:hand2 EDE-p2a-EGFP)*^*bns716*^, *Tg(hand2 eh22:hand2 P-p2a-EGFP)*^*bns717*^ and *Tg(hand2 eh22:hand2 AA-p2a-EGFP)*^*bns718*^ lines, we cloned the *hand2-p2a-EGFP*, *hand2 EDE-p2a-EGFP*, *hand2 P-p2a-EGFP* and *hand2 AA-p2a-EGFP* sequences into a Tol2 backbone downstream of the 752-bp *hand2* enhancer *(hand2 eh22)*.

To generate the *Tg(myl7:pdgfra-p2a-EGFP)*^*bns662*^ line, we cloned the *pdgfra-p2a-EGFP* sequences into a Tol2 backbone downstream of 800 bp of the *myl7* promoter.

To generate the *Tg(hand2 eh22:pdgfra-p2a-EGFP)*^*bns666*^ line, we cloned the *pdgfra-p2a-EGFP* sequences into a Tol2 backbone downstream of 752 bp of the *hand2* enhancer (*hand2 eh22*).

To generate the *Tg(pdgfra enhancer:EGFP)*^*bns741*^, we cloned a 616-bp *pdgfra* enhancer sequence into a Tol2 backbone.

Cloning was performed using In Fusion Cloning (Takara Bio). The constructs were injected into AB embryos at the one-cell stage (20 pg per embryo) together with *Tol2* mRNA (25 pg per embryo) to establish the lines. F_0_ embryos positive for EGFP fluorescence were raised to adulthood and then screened for founder animals. The founders (two for each transgenic line) were further outcrossed to raise the F_1_ generation.

### Molecular genotyping

Genotyping of the *hand2 s6* allele was performed as previously described^14^. *hand2 FLD* mutants were genotyped by PCR using a forward primer in the 5′ UTR region upstream of the deletion (*hand2* fw1: ACAGAGTGAATCAGGCTGCG) and a reverse primer downstream of the deletion (*hand2* rv1: GACGGAAGTGCACTGAATGG), leading to a 353-bp product. To detect the WT allele, we used a forward primer in the 5′ UTR region upstream of the deletion (*hand2* fw2: GAATTCGCCTGCTTCTACAGAGTG) and a reverse primer placed inside the deleted region (*hand2* rv2: CTGCAAATATCCATCACGAGAA), leading to a 194-bp product. High-resolution melt (HRM) analysis was used to genotype the *hand2 Δ27*^*bns540*^ and *hand2 Δ3*^*bns603*^ mutants. *hand2 Δ27* HRM fw: CCGCGGACAGTGAAACGTA; *hand2 Δ27* HRM rv: CCCTGAGTTCTGCAAAGGCG; *hand2 Δ3* HRM fw: ACGGCAAACCGAAAGGAGAG; *hand2 Δ3* HRM rv: TTTGGATAGCTTCGTATCCGC.

*Tg(myl7:pdgfra-p2a-EGFP)* and *Tg(hand2 eh22:pdgfra-p2a-EGFP)* were genotyped by PCR combining a forward primer in the coding region of *pdgfra* (*pdgfra* GT fw: CTAGCAGCTCCACGACCAAGCGTGA) with one reverse primer in the coding region of *EGFP* (*EGFP* rv: CTCGCCGGACACGCTGAACTTGT) to detect the transgene. Genotyping of the *npas4l Pt(npas4l-p2A-Gal4-VP16)*^*bns313*^ allele was performed as previously described^47^.

### Wholemount immunostaining

Whole-mount immunostaining was performed as previously described^76^. Embryos were fixed in 4% paraformaldehyde overnight at 4 °C. The fixative was substituted with PBS/0.1% Tween. The blocking step preceding primary antibody incubation was performed in PBDT (PBS, 1% BSA, 1% DMSO, 0.5% Triton X-100) supplemented with 5% goat serum. Primary antibody incubations were performed overnight at 4 °C at the following concentrations: GFP (1:400, chicken, Aves Labs) and DsRed (1:200, rabbit; Takara Bio). All secondary antibodies were incubated overnight at 4 °C at a concentration of 1:500: Alexa Fluor 568 and Alexa Fluor 488 (Thermo Fisher Scientific). Embryos were incubated with 1 µg ml^−1^ DAPI with the secondary antibody.

### Wholemount RNA in situ hybridization

Digoxigenin-labeled probes were transcribed in vitro from a linearized plasmid (for *pdgfra*^51^) or PCR products by using SP7 polymerase (Promega) and a digoxigenin RNA labeling kit (Roche).

in situ hybridization was performed as described previously^77^. Embryos were imaged using an inverted microscope.

### mESC cell culture and differentiation

V6.5 and E14 mESCs (gifts from T. Braun, Max Planck Institute for Heart and Lung Research) were cultured on 0.1% gelatin-coated plates in ESC medium consisting of DMEM (Gibco) supplemented with 15% FBS (Sigma, F2492), 1 mM sodium pyruvate (Gibco), 0.1 mM nonessential amino acids (Gibco), 2 mM l-glutamine (Gibco), 100 μM β-mercaptoethanol (Sigma), 100 U ml^−1^ penicillin and 100 μg ml^−1^ streptomycin (Hyclone), 1,000 U ml^−1^ recombinant leukemia inhibitory factor (Millipore) and 2i (GSK3 inhibitor: CHIR99021 and MEK inhibitor: PD0325901).

V6.5 and E14 mESCs were differentiated in cardiac differentiation medium as described below. In brief, differentiation through hanging droplets was initiated following ESC dissociation and suspension at 50,000 cells per ml in DMEM with 15% FBS without leukemia inhibitory factor and 2i in 20-μl drops. Two days after droplet formation, embryoid bodies (EBs) were transferred in suspension onto dishes coated with poly-HEMA (2-hydroxyethyl methacrylate) (Sigma P3932). After another 96 h, EBs were plated on gelatin-coated dishes in cardiac differentiation medium (StemPro-34 SF, Invitrogen) supplemented with 5 ng ml^−1^ VEGF (R&D Systems, 293-VE), 10 ng ml^−1^ bFGF (R&D Systems, 233-FB), 12.5 ng ml^−1^ FGF10 (R&D Systems, 345-FG), 2.5 mM XAV939 (Cayman Chemical, 13596), 1 mM ascorbic acid (Sigma, A4403) and 2 mM GlutaMAX. Beating cells were visible 30–48 h after the EBs were plated in the cardiac differentiation medium.

### Plasmids and transfection

The coding sequence of full-length mouse *Hand2* (National Center for Biotechnology Information (NCBI) reference sequence: NM_010402.4) was amplified using PrimeSTAR polymerase (Takara Bio) from the complementary DNA (cDNA) of E14 mESCs^78^. Amplified fragments were subcloned into the pSBbi-GP vector (Addgene, 60511). For transfection, EBs were plated in 12-well plates at a density of approximately 80 EBs per well 1 day before transfection. Transfection was performed using Lipofectamine 3000 (Invitrogen) mixed with appropriate plasmids according to the manufacturer’s instructions.

The endoribonuclease-prepared siRNA of *Hand2* (EMU161511) and siRNA Universal Negative Control #1 (SIC001) were purchased from Sigma-Aldrich. Transfection was performed using Lipofectamine RNAiMAX (Invitrogen) mixed with appropriate siRNA according to the manufacturer’s instructions.

### Confocal microscopy imaging

Embryos were embedded on their side in 1% low-melting agarose/egg water. Living embryos were anesthetized with 0.01% tricaine before embedding and kept under anesthesia during the procedure. All experiments on living embryos were nonrecovery experiments. For genotyping, the anesthetized embryos were taken out of the agarose, exposed to heat briefly and lysed using 50 mM NaOH for 10 min at 95 °C.

All confocal images were acquired using a Zeiss LSM 800 Examiner confocal microscope. The images were acquired and processed using the ZenBlue software package. Only linear adjustments were used; acquisition parameters were kept constant throughout the imaging whenever possible. The confocal microscopy data presented in this article were used for qualitative and quantitative analyses. To analyze the number of cardiomyocytes, we used *Tg(myl7:EGFP)*^+^ embryos to perform whole-mount immunostaining and acquire a *z* stack of confocal images on a Zeiss LSM 800 Examiner. The Spots function in Imaris was used to count the number of EGFP^+^ cells in three-dimensional reconstructions (the lack of a cardiomyocyte nuclear marker made this quantification somewhat challenging).

### Lightsheet time-lapse imaging

Embryos at 14 hpf were sorted for fluorescence and mounted in their chorions with 0.5% agarose into a cobweb holder. No tricaine was used; the temperature was kept stable at 28.5 °C. Stacks were acquired from four different angles and processed as *z* projections.

### Sample preparation and scRNA-seq

We in-crossed *TgBAC(hand2:GFP)*; *hand2 FLD* fish to generate *hand2 FLD* mutant and WT sibling embryos. We collected 100 whole embryos per condition at 24 hpf in cold 1× HBSS (Thermo Fisher Scientific, 88281). We prepared dissociation enzyme 1 (with papain) and enzyme 2 (with thermolysin) according to the manufacturer’s protocol (Thermo Fisher Scientific, 88281). We then mixed 200 µl of enzyme 1 with 10 µl of enzyme 2 and added 100 µl of the mixture to each of the two conditions. We kept the tubes in a 300-rpm shaker at 30 °C for 20 min, quenched the reaction by adding 1 ml DMEM–10% FBS, and finished breaking up the tissues by pipetting up and down. We pelleted the cells by centrifugation at 500*g* for 5 min at 4 °C and replaced the medium with DMEM–10% FBS. Then, we FACS-sorted the cells for GFP fluorescence using an Aria III sorter and DAPI as an indicator for dead cells. The cell suspensions were analyzed using a Moxi cell counter and diluted according to the manufacturer’s protocol to obtain 5,000 single-cell data points per sample. Each sample was run separately on a lane in a Chromium controller with Chromium Next GEM Single Cell 3ʹ Reagent Kits v3.1 (10x Genomics). scRNA-seq library preparation was done using a standard protocol. Sequencing was done on a NextSeq 2000 instrument.

### Single-cell transcriptome analysis

Sequenced raw reads were aligned against the zebrafish genome (danRer10 assembly 101) and counted by STARsolo^79^. Raw counts per gene and cell identifiers were stored in an annotated data format^80^ for all further analysis steps, holding initially 5,054 and 5,667 cells for *hand2 FLD*^+/?^ and *hand2 FLD*^−/−^ samples, respectively. Next, cells were analyzed using the Scanpy framework^81^. Preprocessed counts were used to calculate quality metrics and to estimate cell quality, taking into account ribosomal genes (excluded), mitochondrial content (<50%), number of genes (>300), number of cells per gene (>30) and total gene count (<8,000). In summary, we reduced the cell numbers to 3,836 and 3,900 *hand2* reporter-expressing cells from 24-hpf *hand2 FLD*^+/?^ and *hand2 FLD*^−/−^ sibling embryos, respectively. Following quality control, raw counts per cell were normalized to the median count of all cells and transformed into log space to stabilize the variance. After carrying out a principal component analysis, ten principal components were used for neighbor calculation. We generated low-dimensional UMAP embedding^82^ by using a minimum distance of 0.1 and a spread of 2.5. Clustering was performed using the Leiden algorithm at multiple resolutions. After manual inspections, we selected seven cell clusters, as the corresponding marker genes best reflected the expected cell populations at this resolution. Final data visualization was done with the CellxGene package (10.5281/zenodo.3235020).

### Nuclear extraction and FACS sorting for ChIP–seq and ATAC–seq

Timed matings were set up between *Tg(myl7:EGFP)* adults, and embryos were maintained at 28.5 °C for 24 h from the time of fertilization. Chorions were removed by incubating in 15 ml pronase E at 1 mg ml^−1^ for 10 min with continuous shaking. Pronase E was removed by five washes with 200 ml egg water (60 μg ml^−1^ ocean salt (Red Sea), 3 μM methylene blue). Embryos were then briefly washed in PBT (0.1% Triton X-100 in PBS) and fixed in 0.5% formaldehyde (Carl Roth, #4979.1) in PBS for 15 min. Next, they were washed in PBT–glycine (PBS, 125 mM glycine, 0.1% Triton X-100), washed twice in PBT (PBS, 0.1% Triton X-100) and snap frozen in liquid nitrogen. Nuclei were extracted as described previously^83^; embryos were resuspended in 2 ml cell lysis buffer (10 mM Tris–HCl, pH 7.5, 10 mM NaCl, 0.5% IGEPAL (CA-630, Sigma, I8896)), homogenized on ice for 15 min (dounced 20 times with a loose pestle and 10 times with a tight pestle) and then spun at 2,000*g* for 5 min at 4 °C.

To enhance the fluorescence signal, we stained the nuclei with a GFP antibody (Thermo Fisher Scientific, #G10362) at a 1:100 dilution for 1 h at 4 °C in PBTB (PBS, 0.1% Triton X-100, 5% BSA). Nuclei were washed in 6 ml PBTB and spun at 1,000*g* for 2 min at 4 °C. The secondary antibody, Alexa Fluor 488 (Abcam, #ab150077), was added at 1:100 in PBTB, followed by incubation for 1 h at 4 °C. Nuclei were further stained with DAPI (Scientific Laboratory Supplies, #D9542-1MG), and 20,000 GFP-positive and 20,000 GFP-negative nuclei were sorted using FACS (BD FACS Aria III) and snap frozen.

### ChIP–seq

FACS-sorted GFP-positive and GFP-negative nuclei were lysed for 10 min on ice in nucleus lysis buffer (50 mM Tris–HCl, pH 7.5, 10 mM EDTA, 1% SDS, protease inhibitor cocktail). Two volumes of IP dilution buffer (16.7 mM Tris–HCl, pH 7.5, 167 mM NaCl, 1.2 mM EDTA, 0.01% SDS, protease inhibitor cocktail) were added, and aliquots were sonicated for 16 cycles (30-s on, 30-s off, at a high setting) in a Bioruptor Plus (Diagenode) to achieve a DNA fragment size of <500 bp. ChIP was performed using True MicroChIP Kit (Diagenode, #C01010130) according to the manufacturer’s instructions with modifications as described previously^84^: primary antibody H3K27ac (Abcam, #ab4729) was incubated at 4 °C overnight, and reverse cross-linking was done overnight. The library was prepared using NEXTflex qRNA-Seq Kit v2 (Bioo Scientific, #5130-12, discontinued) according to the instructions for qChIP–seq, and paired-end sequencing (2 × 75 nucleotides) was performed on a NextSeq 500/550 High Output v2 kit for 150 cycles (Illumina, #FC-404-2002, discontinued).

### ATAC–seq

Sorted GFP-positive and GFP-negative nuclei were washed and lysed in 1 ml water and spun at 12,000*g* for 20 min. Tagmentation was performed according to the kit instructions (Illumina, #20034197) at 37 °C for 1 h by gently shaking (300 rpm) in 25 μl TD buffer, 20 μl water and 5 μl TDE1. Next, 50 μl stop buffer (50 mM Tris–HCl, pH 8.0, 100 mM NaCl, 0.1% SDS, 100 mM 100 mM EDTA, pH 8.0, 1 mM spermidine, 0.3 mM spermine, 40 μg ml^−1^ ribonuclease A) was added, and the samples were incubated at 55 °C for 10 min. Then, 3 μl proteinase K (20 mg ml^−1^) was added, followed by incubation at 65 °C overnight. The DNA was then purified using a Qiagen MinElute column. Both samples were amplified for 14 cycles as described previously^85^. DNA fragments smaller than 1 kb were cut from the gel and gel purified. Sequencing (2 × 75 nucleotides) was performed on a NextSeq 500/550 High Output v2 kit for 150 cycles (Illumina, #FC-404-2002, discontinued).

### ChIP–seq data processing

Processing steps were implemented within the Snakemake framework^86^. Unique molecular identifiers were extracted from paired-end reads using UMI-tools^87^ and mapped to the danRer11 genome assembly using Bowtie 2 (with the parameter ‘–X 2000–no-discordant–no-mixed’). Mapped reads were sorted, indexed and converted to .bam format with SAMtools^88^ and then filtered for MAPQ 30 and deduplicated using UMI-tools. Input-subtracted bigwig files for visualizing (–operation subtract–binSize 50–scaleFactorsMethod None–normalizeUsing CPM–smoothLength 250–extendReads) tracks were generated using deepTools^89^.

### ATAC–seq data processing

Adaptors were removed using Flexbar^90^ and mapped to danRer11 using Bowtie 2 (ref. ^91^) (–X 1500–no-discordant–no-mixed). Mapped reads were sorted, indexed, converted to .bam format, filtered for MAPQ 30 and deduplicated using SAMtools^88^. The start sites of reads were extended by 15 bp upstream and 22 bp downstream in a stranded manner using BEDTools^92^, to account for steric hindrance of the transposition reaction^93^. Bigwig signal tracks were generated using deepTools^89^ (–binSize 10–normalizeUsing CPM–smoothLength 50–extendReads 38).

### Footprinting analysis

Paired-end reads in fastq format from ATAC samples for cardiomyocytes and noncardiomyocytes were mapped to the danRer11 genome with STAR version 2.7.3a^79^. The resulting .bam files were used for genomic footprinting analysis, performed with the TOBIAS tool^41^ using the 2020 JASPAR vertebrate database for transcription factor motifs.

### Transcriptomic analysis

For RNA-seq, total RNA was isolated using a miRNeasy Micro kit (Qiagen) with a low-input DNase protocol. RNA and library preparation integrity was verified with LabChip GX Touch 24 (PerkinElmer). Approximately 1 ng total RNA was used as starting material for the SMART-Seq HT kit (Takara Bio). Sequencing was performed on the NextSeq 2000 instrument (Illumina) using a P3 flow cell with a 1 × 72-bp single-end setup. The resulting raw reads were assessed for quality, adaptor content and duplication rates with FastQC (http://www.bioinformatics.babraham.ac.uk/projects/fastqc). Trimmomatic version 0.39 was used to trim reads after a quality drop below a mean of Q20 in a window of 20 nucleotides. Only reads between 15 and 75 nucleotides were cleared for further analyses. Trimmed and filtered reads were aligned versus the Ensembl zebrafish genome version danRer11 (ensemble release 104) using STAR version 2.7.10a with the parameter ‘outFilterMismatchNoverLmax 0.1’ to increase the maximum ratio of mismatches to mapped length to 10%. The number of reads aligning to genes was counted with the featureCounts version 2.0.2 tool from the Subread package. Only reads mapping at least partially inside exons were admitted and aggregated per gene. Reads overlapping multiple genes or aligning to multiple regions were excluded. DEGs were identified using DESeq2 version 1.30.1. Only genes with a minimum fold-change value of ±1.5 (log_2_ ± 0.59), a maximum Benjamini–Hochberg corrected *P* value of 0.05, a minimum combined mean of five reads and −0.585 < log_2_(fold change) > 0.585 were deemed to be significantly differentially expressed. DEGs were split into upregulated and downregulated genes. For the *hand2* mutant versus WT comparison, they consisted of 1,926 downregulated genes and 1,828 upregulated genes. For the *hand2* OE versus control comparison, they consisted of 525 downregulated genes and 812 upregulated genes. For the KOBAS^94^ gene set enrichment analysis, DEGs were split into upregulated and downregulated genes. Significant gene set enrichment was defined by false discovery rate, and the top 10 gene sets or enriched pathways were plotted (dashed line: *P* = 0.05).

### qPCR analysis

RNA was extracted with standard TRIzol–chloroform extraction. Approximately 500–1,000 ng RNA was used to synthesize cDNA using the Maxima first-strand cDNA kit (Thermo Fisher Scientific). For all experiments, DyNAmo ColorFlash SYBR Green qPCR Mix (Thermo Fisher Scientific) was used on a CFX Connect real-time system (Bio-Rad). All reactions were performed in technical triplicates and from three or more biological replicates. Gene expression values were normalized using the zebrafish housekeeping gene *rpl13a* or the mouse housekeeping gene *Gapdh*, and fold changes were calculated using the 2^−ΔΔCt^ method. Primers are listed in Supplementary Table 1.

### Western blot analysis

We collected 100 embryos at 14 and 22 hpf, deyolked them and then lysed them in the CoIP buffer (50 mM Tris–HCl, pH 7.5, 150 mM NaCl, 10% glycerol, 2 mM EDTA, 0.5% NP-40 and protease inhibitors). Protein concentrations were determined using a Pierce BCA protein assay kit (Thermo Fisher Scientific) according to the manufacturer’s instructions. Total proteins (80 μg) were separated on a 4–12% Bis–Tris NuPAGE gel (Invitrogen, NP0335) and run at 110 V for 1.5 h. Protein transfer was done for 1.5 h at 110 V onto a 0.2-μm nitrocellulose membrane (Invitrogen, LC2000). The membrane was blocked in 5% milk–PBT (PBS + 0.1% Tween 20) for 1 h and incubated overnight at 4 °C with mouse anti-FLAG M2 (1:1,000, Sigma, F1804) or mouse anti-β-actin (1:1,000, Sigma, A5441) primary antibodies in 5% milk–PBT. Anti-mouse (1:5,000, Abcam, ab97023) HRP-linked secondary antibodies were used and incubated for 2 h at room temperature in 5% milk–PBT. Chemiluminescent detection was performed using the SuperSignal West Pico PLUS chemiluminescent substrate kit (Thermo Fisher Scientific, 34577) on a Bio-Rad ChemiDoc MP imaging system.

### Coimmunoprecipitation

We collected 400 embryos at 14 hpf. After deyolking the embryos, we lysed them in the CoIP buffer (50 mM Tris–HCl, pH 7.5, 150 mM NaCl, 1 mM EDTA, 1% Triton X-100 and protease inhibitors). Protein concentrations were determined using a Pierce BCA protein assay kit (Thermo Fisher Scientific) according to the manufacturer’s instructions. An 800-μg amount of the soluble fraction was taken for immunoprecipitation using 25 μl anti-FLAG M2 magnetic beads (Sigma, M8823) in a total of 800 μl CoIP fresh buffer and incubated on the rotor at 4 °C overnight. The beads were pulled aside with the magnet and washed three times with freshly prepared 1× TBS supplemented with protease inhibitors and subsequently processed for MS in the manner described below.

### MS and data analysis

Washed beads were mixed with 30 μl digestion buffer (6 M urea, 2 M thiourea), reduced with 10 mM dithiothreitol for 30 min at room temperature and alkylated with 55 mM iodoacetamide for 30 min at room temperature in the dark. Each sample was diluted with 60 μl of 0.1 M TEAB and digested with 0.5 μg trypsin overnight. Digested proteins were desalted using a C18 stage tip. Desalted peptides were measured by liquid chromatography–MS/MS. Quantitative analyses were performed on an Orbitrap Q-exactive HF MS system (Thermo Fisher Scientific) coupled to an EASY-nLC capillary nanochromatography system (Thermo Fisher Scientific). Desalted peptides were separated on an in-house-made capillary column (150 mm × 1.7 µm × 75 µm) packed with ReproSil-Pur 120 C18-AQ resin (Dr. Maisch). The mobile phases were A (2% acetonitrile, 0.1% formic acid) and B (90% acetonitrile, 0.1% formic acid). Peptides were separated using a 230-min acetonitrile gradient at room temperature. The mass spectrometer was operated in positive electrospray ionization mode, and MS/MS data were collected in data-dependent analysis mode with a resolution of 60,000 for precursor mass spectra and 15,000 for tandem mass spectra. Normalized collision energy was set to 28, and exclusion time was set to 30 s. Collected data were processed using MaxQuant software. Data were filtered such that potential contaminants, reverse sequences, proteins identified only by site and proteins with a high proportion of missing values were removed. Intensities were converted to a log_2_ scale, and missing values were calculated using the MNAR (missing not at random) method. The setting of significant protein filtering criteria was as follows: adjusted *P*-value cutoff = 0.05 and log_2_(fold change) cutoff = 1. A *t* test was used for correcting the false discovery rate.

### Evolutionary analysis of *HAND1*

To identify the orthologs of human HAND1 (SwissProtID: O96004) across a broad array of species, including 4 mammals, 3 crocodiles, 2 amphibians and 12 teleosts, we downloaded genomic data from the NCBI Genome database as of February 2024 (https://ftp.ncbi.nlm.nih.gov/genomes/refseq/). To create the database of the mentioned genomes and evaluate the pairwise protein feature architecture similarities between the HAND1 protein and the listed orthologs, we performed the analysis following the method outlined by Koestler et al.^95^, using the FAS (feature architecture similarity) tool (https://github.com/BIONF/FAS). The resultant phylogenetic profiles and protein feature architecture data were visualized and analyzed using PhyloProfile^96^.

### Statistical analysis

All statistical analyses were performed in GraphPad Prism version 8.4. Western blot experiments were repeated three to four times and gave similar results.

### Reporting summary

Further information on research design is available in the Nature Portfolio Reporting Summary linked to this article.

## Supplementary information

Supplementary Information Supplementary Figs. 1 and 2, figure legends and reference.

Reporting Summary

Supplementary Tables Supplementary Table 1. *C*_t_ values of mRNA levels from RT–qPCR experiments and primers used in this study. Supplementary Table 2. List of differentially expressed genes in each cell cluster from the scRNA-seq in this study. Supplementary Table 3. List of the interacting proteins in each relevant sample of this study.

Supplementary Video 1 Time-lapse imaging of *TgBAC(hand2:EGFP)* (blue) and *Tg(kdrl:mCherry)* (magenta) expression in a *hand2 FLD*^+/+^ embryo from 16 to 24 hpf.

Supplementary Video 2 Time-lapse imaging of *TgBAC(hand2:EGFP)* (blue) and *Tg(kdrl:mCherry)* (magenta) expression in a *hand2 FLD*^−/−^ embryo (a sibling of the *hand2 FLD*^+/+^ embryo shown in Supplementary Video 1) from 16 to 24 hpf.

Supplementary Video 3 Video of mESC-derived cardiac cells.

## Source data

Source Data Extended Data Fig. 5 Uncropped and unprocessed scans for Extended Data Fig. 5e (panel 1).

## Data Availability

The scRNA-seq and RNA-seq data reported in this paper have been deposited in the Gene Expression Omnibus (GEO) database under accession nos. GSE241971 (scRNA-seq, ChIP–seq and ATAC–seq) and GSE241049 (RNA-seq). Source data are provided with this paper.
