# Supplementary material for: PDGFRA is a conserved HAND2 effector during early cardiac development
Source: Nat Cardiovasc Res. 2024 Dec 10;3(12):1531–48. doi: 10.1038/s44161-024-00574-1 (PMC11634778; doi:10.1038/s44161-024-00574-1)
Supplement: Supplementary file 1 — Supplementary Figs. 1 and 2, figure legends and reference. [file 44161_2024_574_MOESM1_ESM.pdf]

nature cardiovascular research

Supplementary information https://doi.org/10.1038/s44161-024-00574-1

PDGFRA is a conserved HAND2 effector during early cardiac development

In the format provided by the authors and unedited

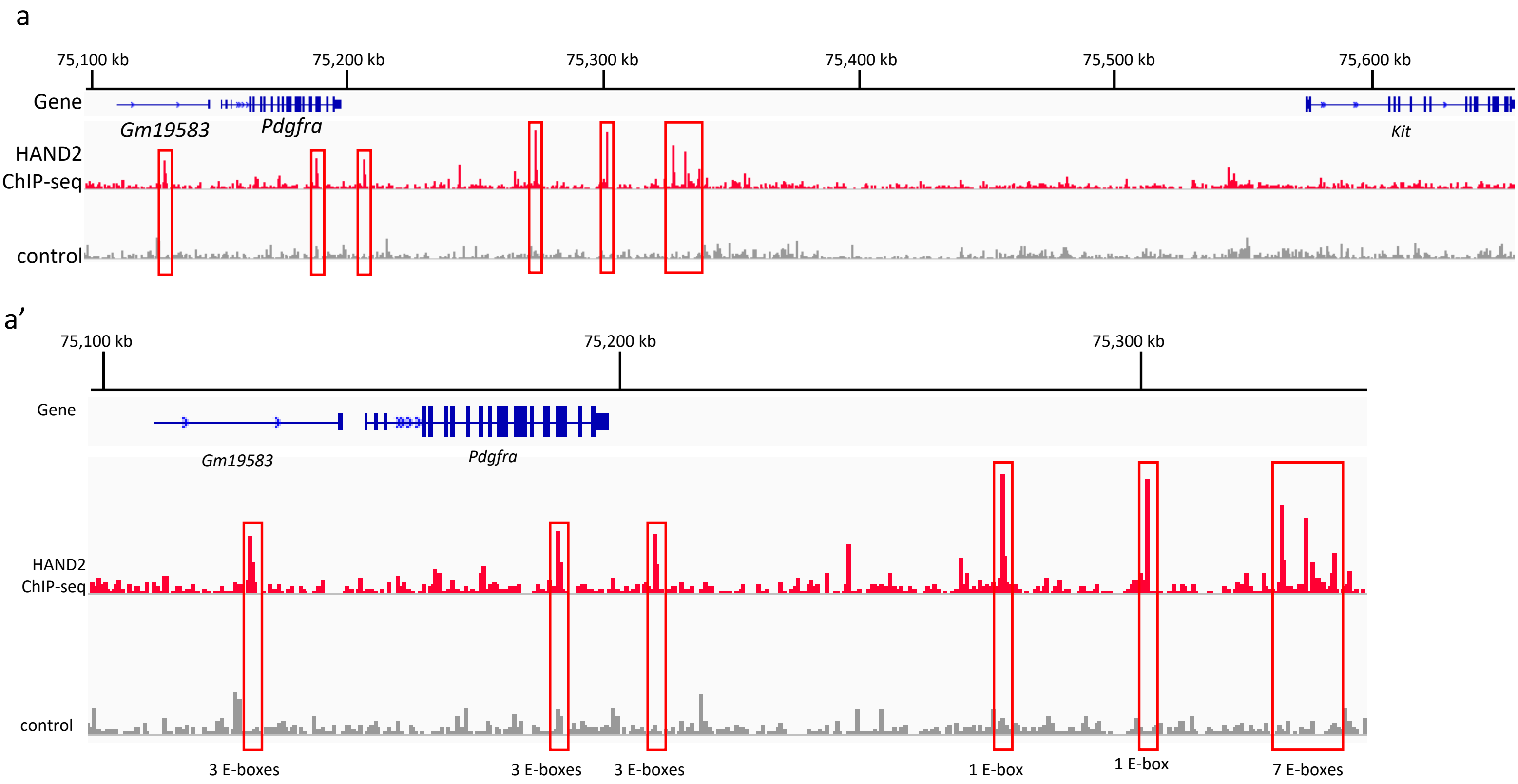
a
75,100 kb
75,200 kb
75,300 kb
75,400 kb
75,500 kb
75,600 kb
Gene
Gm19583
Pdgfra
Kit
HAND2
ChIP-seq
control
a’
75,100 kb
75,200 kb
75,300 kb
Gene
Gm19583
Pdgfra
HAND2
ChIP-seq
control
3 E-boxes
3 E-boxes
3 E-boxes
1 E-box
1 E-box
7 E-boxes

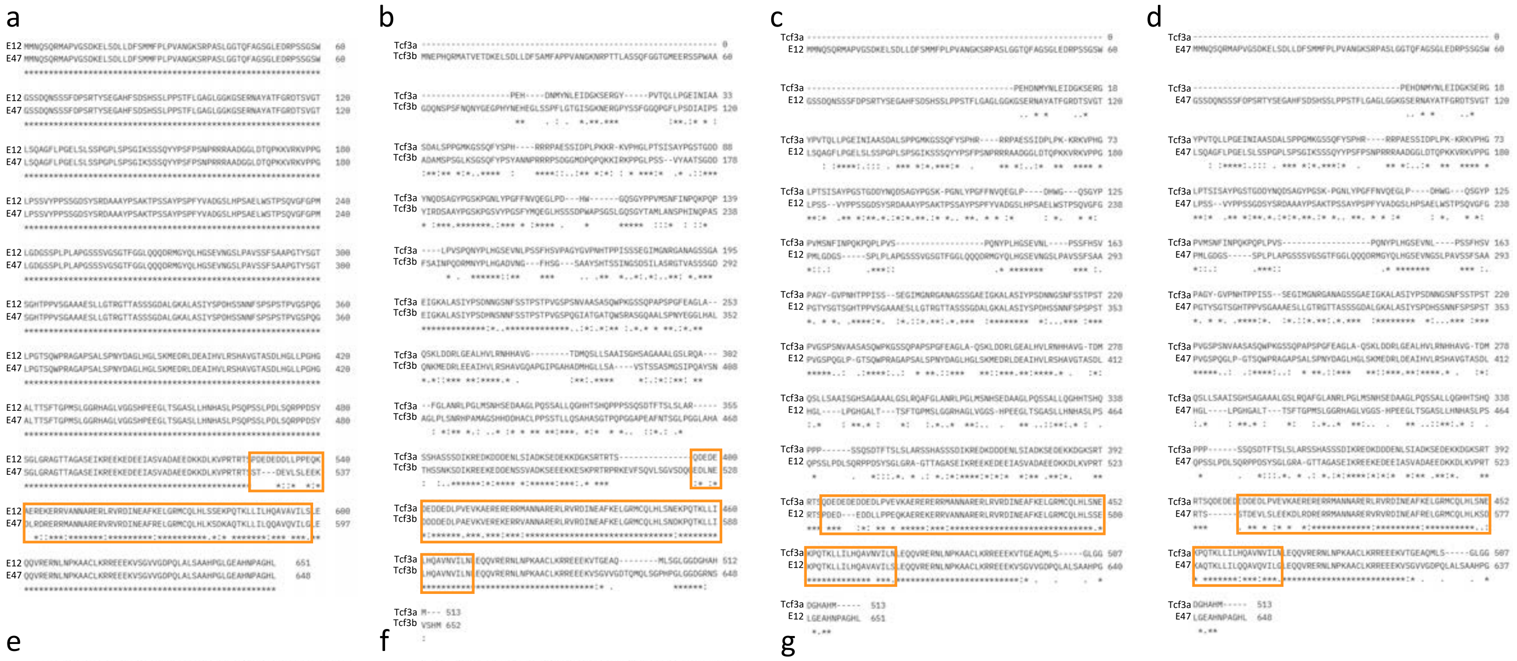

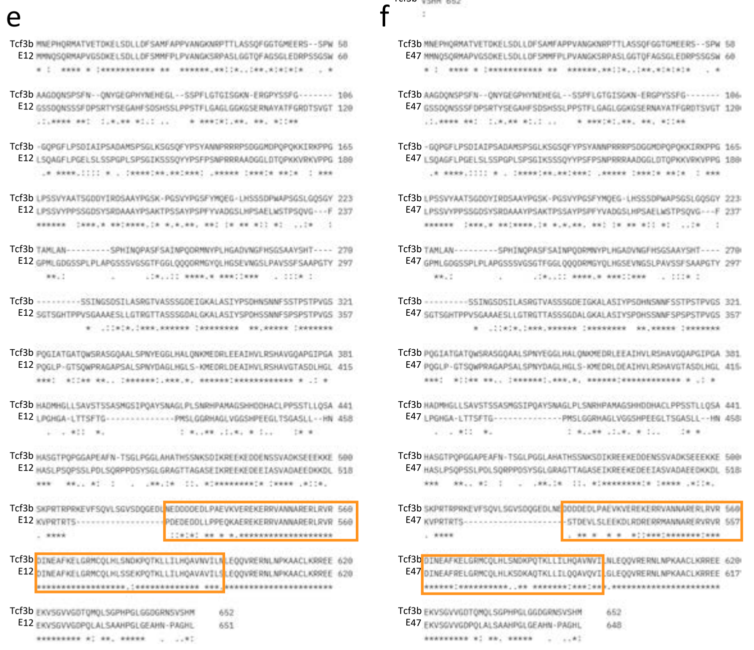

g

Protein A	Protein B	Protein sequence similarity (%)
E12	E47	96.47
Tcf3a	Tcf3b	52.98
Tcf3a	E12	48.06
Tcf3a	E47	46.36
Tcf3b	E12	52.30
Tcf3b	E47	51.01

Supplementary Fig 1: Putative HAND2-binding sites in the mouse *Pdgfra* locus. a, a', Genome browser view showing HAND2 ChIP-seq peaks enriched in E10.25–10.5 mouse hearts at the *Pdgfra* locus (data analyzed from GSE55707[1]). Red boxes: HAND2-binding sites.

Supplementary Fig 2: Amino acid sequence of zebrafish Tcf3a and Tcf3b. a, Amino acid sequence alignment of zebrafish Tcf3a and Tcf3b generated through Clustal Omega; the orange box encompasses the bHLH domain. b, Modeling of the structure of Tcf3a and Tcf3b by Alphafold2.

1 Laurent, F. *et al.* HAND2 Target Gene Regulatory Networks Control Atrioventricular Canal and Cardiac Valve Development. *Cell Rep* **19**, 1602-1613, doi:10.1016/j.celrep.2017.05.004 (2017).
