# Supplementary material for: PDGFRA is a conserved HAND2 effector during early cardiac development
Source: Nat Cardiovasc Res. 2024 Dec 10;3(12):1531–48. doi: 10.1038/s44161-024-00574-1 (PMC11634778; doi:10.1038/s44161-024-00574-1)
Supplement: Supplementary file 2 — Reporting Summary [file 44161_2024_574_MOESM2_ESM.pdf]

nature portfolio

Corresponding author(s): Didier Stainier

Last updated by author(s): Oct 23, 2024

Reporting Summary

Nature Portfolio wishes to improve the reproducibility of the work that we publish. This form provides structure for consistency and transparency in reporting. For further information on Nature Portfolio policies, see our Editorial Policies and the Editorial Policy Checklist.

Statistics

For all statistical analyses, confirm that the following items are present in the figure legend, table legend, main text, or Methods section.

n/a	Confirmed	
☐	☒	The exact sample size (*n*) for each experimental group/condition, given as a discrete number and unit of measurement
☐	☒	A statement on whether measurements were taken from distinct samples or whether the same sample was measured repeatedly
☐	☒	The statistical test(s) used AND whether they are one- or two-sided<br>*Only common tests should be described solely by name; describe more complex techniques in the Methods section.*
☒	☐	A description of all covariates tested
☐	☒	A description of any assumptions or corrections, such as tests of normality and adjustment for multiple comparisons
☐	☒	A full description of the statistical parameters including central tendency (e.g. means) or other basic estimates (e.g. regression coefficient) AND variation (e.g. standard deviation) or associated estimates of uncertainty (e.g. confidence intervals)
☐	☒	For null hypothesis testing, the test statistic (e.g. *F*, *t*, *r*) with confidence intervals, effect sizes, degrees of freedom and *P* value noted<br>*Give P values as exact values whenever suitable.*
☒	☐	For Bayesian analysis, information on the choice of priors and Markov chain Monte Carlo settings
☒	☐	For hierarchical and complex designs, identification of the appropriate level for tests and full reporting of outcomes
☐	☒	Estimates of effect sizes (e.g. Cohen's *d*, Pearson's *r*), indicating how they were calculated

Our web collection on statistics for biologists contains articles on many of the points above.

Software and code

Policy information about availability of computer code

Data collection	Zen blue, 2.3 lite was used for microscopy imaging; Bio-Rad CFX Manager, 3.1 was used for RT-qPCR; NIS-Elements BR, 4.30.60 was used for in situ images.
Data analysis	Prism GraphPad, 2022 was used to determine the p values and perform all statistical analysis. Imaris, 10.0.0; ImarisFile Converter, 10.0.0 0) was used for 3-D surface rendering. CellxGene package (doi:10.5281/zenodo.3235020) was used to visualize scRNA-seq data.

For manuscripts utilizing custom algorithms or software that are central to the research but not yet described in published literature, software must be made available to editors and reviewers. We strongly encourage code deposition in a community repository (e.g. GitHub). See the Nature Portfolio guidelines for submitting code & software for further information.

Data

Policy information about availability of data

All manuscripts must include a data availability statement. This statement should provide the following information, where applicable:

- Accession codes, unique identifiers, or web links for publicly available datasets
- A description of any restrictions on data availability
- For clinical datasets or third party data, please ensure that the statement adheres to our policy

GSE241971 (scRNA-seq, ChIP-seq, and ATAC-seq), GSE241049 (RNA-seq). All the raw reads were aligned against the zebrafish genome (danRer11).

Research involving human participants, their data, or biological material

Policy information about studies with human participants or human data. See also policy information about sex, gender (identity/presentation), and sexual orientation and race, ethnicity and racism.

Reporting on sex and gender	N/A
Reporting on race, ethnicity, or other socially relevant groupings	N/A
Population characteristics	N/A
Recruitment	N/A
Ethics oversight	N/A

Note that full information on the approval of the study protocol must also be provided in the manuscript.

Field-specific reporting

Please select the one below that is the best fit for your research. If you are not sure, read the appropriate sections before making your selection.

☒ Life sciences ☐ Behavioural & social sciences ☐ Ecological, evolutionary & environmental sciences

For a reference copy of the document with all sections, see nature.com/documents/nr-reporting-summary-flat.pdf

Life sciences study design

All studies must disclose on these points even when the disclosure is negative.

Sample size	In addition, Sample size was determined based on our previous experience and the work of ours and other groups working on zebrafish heart development:<br>Jimenez-Amilburu, V. et al. Development. (2019).<br>Uribe, V. et al. Development. (2018).<br>Rasouli, S. J. et al. Nat Commun. (2017).<br>Peralta, M. et al. Curr Biol. (2013).<br>Vermot, J. et al. PLoS Biol. (2009).<br>Bornhorst, D. et al. Nat Commun. (2019).<br>Auman, H. J. et al. PLoS Biol. (2007).<br>Duchemin, A. L. et al. Elife. (2019)<br>scale bar in Imaris, 10.0.0; Zen blue, 2.3 lite; NIS-Elements BR, 4.30.60 were used to determine the size of samples.
Data exclusions	no data exclusion.
Replication	All experiments were repeated independently at least three times using different batches of animals on different days, and got similar results.
Randomization	After selecting embryos for proper morphology and screening them for the relevant fluorescent signals in the heart , the samples were randomly allocated to different experimental groups in each experiment.
Blinding	Whenever possible, blinding was performed in data collection and analysis.<br>In some experiments, when embryos had very obvious phenotype, blinding was not possible as the same investigator processed the samples and collected the data. For example, when we compared the cardia bifida embryos with the linear heart tube embryos. To address potential bias, we ensured that all researchers involved in performing experiments and data analysis underwent thorough training and adhered to standardized protocols. In addition, quantifications were done in a consistent manner every time and thus even if there is some technical bias, it is the same throughout all the samples.

Behavioural & social sciences study design

All studies must disclose on these points even when the disclosure is negative.

Study description	*Briefly describe the study type including whether data are quantitative, qualitative, or mixed-methods (e.g. qualitative cross-sectional, quantitative experimental, mixed-methods case study).*
Research sample	*State the research sample (e.g. Harvard university undergraduates, villagers in rural India) and provide relevant demographic information (e.g. age, sex) and indicate whether the sample is representative. Provide a rationale for the study sample chosen. For studies involving existing datasets, please describe the dataset and source.*

Sampling strategy	*Describe the sampling procedure (e.g. random, snowball, stratified, convenience). Describe the statistical methods that were used to predetermine sample size OR if no sample-size calculation was performed, describe how sample sizes were chosen and provide a rationale for why these sample sizes are sufficient. For qualitative data, please indicate whether data saturation was considered, and what criteria were used to decide that no further sampling was needed.*
Data collection	*Provide details about the data collection procedure, including the instruments or devices used to record the data (e.g. pen and paper, computer, eye tracker, video or audio equipment) whether anyone was present besides the participant(s) and the researcher, and whether the researcher was blind to experimental condition and/or the study hypothesis during data collection.*
Timing	*Indicate the start and stop dates of data collection. If there is a gap between collection periods, state the dates for each sample cohort.*
Data exclusions	*If no data were excluded from the analyses, state so OR if data were excluded, provide the exact number of exclusions and the rationale behind them, indicating whether exclusion criteria were pre-established.*
Non-participation	*State how many participants dropped out/declined participation and the reason(s) given OR provide response rate OR state that no participants dropped out/declined participation.*
Randomization	*If participants were not allocated into experimental groups, state so OR describe how participants were allocated to groups, and if allocation was not random, describe how covariates were controlled.*

Ecological, evolutionary & environmental sciences study design

All studies must disclose on these points even when the disclosure is negative.

Study description	*Briefly describe the study. For quantitative data include treatment factors and interactions, design structure (e.g. factorial, nested, hierarchical), nature and number of experimental units and replicates.*
Research sample	*Describe the research sample (e.g. a group of tagged Passer domesticus, all Stenocereus thurberi within Organ Pipe Cactus National Monument), and provide a rationale for the sample choice. When relevant, describe the organism taxa, source, sex, age range and any manipulations. State what population the sample is meant to represent when applicable. For studies involving existing datasets, describe the data and its source.*
Sampling strategy	*Note the sampling procedure. Describe the statistical methods that were used to predetermine sample size OR if no sample-size calculation was performed, describe how sample sizes were chosen and provide a rationale for why these sample sizes are sufficient.*
Data collection	*Describe the data collection procedure, including who recorded the data and how.*
Timing and spatial scale	*Indicate the start and stop dates of data collection, noting the frequency and periodicity of sampling and providing a rationale for these choices. If there is a gap between collection periods, state the dates for each sample cohort. Specify the spatial scale from which the data are taken*
Data exclusions	*If no data were excluded from the analyses, state so OR if data were excluded, describe the exclusions and the rationale behind them, indicating whether exclusion criteria were pre-established.*
Reproducibility	*Describe the measures taken to verify the reproducibility of experimental findings. For each experiment, note whether any attempts to repeat the experiment failed OR state that all attempts to repeat the experiment were successful.*
Randomization	*Describe how samples/organisms/participants were allocated into groups. If allocation was not random, describe how covariates were controlled. If this is not relevant to your study, explain why.*
Blinding	*Describe the extent of blinding used during data acquisition and analysis. If blinding was not possible, describe why OR explain why blinding was not relevant to your study.*

Did the study involve field work? ☐ Yes ☐ No

Field work, collection and transport

Field conditions	*Describe the study conditions for field work, providing relevant parameters (e.g. temperature, rainfall).*
Location	*State the location of the sampling or experiment, providing relevant parameters (e.g. latitude and longitude, elevation, water depth).*
Access & import/export	*Describe the efforts you have made to access habitats and to collect and import/export your samples in a responsible manner and in compliance with local, national and international laws, noting any permits that were obtained (give the name of the issuing authority, the date of issue, and any identifying information).*
Disturbance	*Describe any disturbance caused by the study and how it was minimized.*

Reporting for specific materials, systems and methods

We require information from authors about some types of materials, experimental systems and methods used in many studies. Here, indicate whether each material, system or method listed is relevant to your study. If you are not sure if a list item applies to your research, read the appropriate section before selecting a response.

Materials & experimental systems

n/a	Involved in the study
☐	☒ Antibodies
☐	☒ Eukaryotic cell lines
☒	☐ Palaeontology and archaeology
☐	☒ Animals and other organisms
☒	☐ Clinical data
☒	☐ Dual use research of concern
☒	☐ Plants

Methods

n/a	Involved in the study
☐	☒ ChIP-seq
☐	☒ Flow cytometry
☒	☐ MRI-based neuroimaging

Antibodies

Antibodies used

anti-GFP at 1:500 (chicken, GFP-1010, AvesLab); anti-FLAG at 1:2000 (mouse, F1804, Sigma); anti--β-actin at 1:2000 (mouse, A5441, Sigma); anti-H3K27ac at 2 μg for 25 μg chromatin (Rabbit, ab4729, Abcam); anti-GFP at 1:100 (Rabbit, G10362, Thermo Fisher)

Validation

All antibodies used in this study were commerically available.
anti-GFP, https://www.aveslabs.com/products/anti-green-fluoreseent-protein-antibody-gfp.
Manufacturer: Chickens were immunized with purified recombinant green fluorescent protein (GFP) emulsified in Freund's adjuvant.
Reference: PMID: 34732708

anti-FLAG, https://www.sigmaaldrich.com/DE/en/product/sigma/f1804?srsltid=AfmBOorMa-I1ujmqtjpnzvcXXOGdG0nE7ntJl2tCfdTpAnzI8MU3itNe&icid=sharepdp-clipboard-copy-productdetailpage
Reference: PMID: 8024796

anti--β-actin, https://www.sigmaaldrich.com/DE/en/product/sigma/a5441?icid=sharepdp-clipboard-copy-productdetailpage
Reference: PMID: PMID: 38194447

anti-H3K27ac, https://www.abcam.com/en-us/products/primary-antibodies/histone-h3-acetyl-k27-antibody-chip-grade-ab4729#tab=datasheet&application=chip&drawerView=quickview
Reference: PMID: 35810562

anti-GFP, https://www.thermofisher.com/antibody/product/GFP-Antibody-Recombinant-Monoclonal/G10362
Reference: PMID: 36114190

Eukaryotic cell lines

Policy information about cell lines and Sex and Gender in Research

Cell line source(s)

E14; V6.5 (kind gifts from Dr. Thomas Braun from the Max Planck Institute for Heart and Lung Research)

Authentication

Smith AG. Culture and differentiation of embryonic stem cells. Journal of tissue culture methods, (1991).

Mycoplasma contamination

All these lines were tested negative for mycoplasma contamination.

Commonly misidentified lines
(See ICLAC register)

Name any commonly misidentified cell lines used in the study and provide a rationale for their use.

Palaeontology and Archaeology

Specimen provenance

Provide provenance information for specimens and describe permits that were obtained for the work (including the name of the issuing authority, the date of issue, and any identifying information). Permits should encompass collection and, where applicable, export.

Specimen deposition

Indicate where the specimens have been deposited to permit free access by other researchers.

Dating methods

If new dates are provided, describe how they were obtained (e.g. collection, storage, sample pretreatment and measurement), where they were obtained (i.e. lab name), the calibration program and the protocol for quality assurance OR state that no new dates are

provided.

☐ Tick this box to confirm that the raw and calibrated dates are available in the paper or in Supplementary Information.

Ethics oversight	*Identify the organization(s) that approved or provided guidance on the study protocol, OR state that no ethical approval or guidance was required and explain why not.*

Note that full information on the approval of the study protocol must also be provided in the manuscript.

Animals and other research organisms

Policy information about studies involving animals; ARRIVE guidelines recommended for reporting animal research, and Sex and Gender in Research

Laboratory animals	Zebrafish (Danio rerio), strain: Tüb/AB, adult fish (both male and females, less than 2 years old) were used in the study. Most of the experiments were done on embryos or larvae and at this stage, sex is not specified.
Wild animals	The study did not involve wild animals
Reporting on sex	Zebrafish (Danio rerio), strain: Tüb/AB, adult fish (both male and females) were used in the study. Most of the experiments were done on embryos or larvae and at this stage, sex is not specified.
Field-collected samples	The study did not involve samples collected from the field
Ethics oversight	All zebrafish husbandry was performed under standard conditions in accordance with institutional (Max Planck Gesellschaft) and national (German) ethical and animal welfare regulations. All procedures performed on animals were approved by the Tierschutzkommission of the administrative district Darmstadt.

Note that full information on the approval of the study protocol must also be provided in the manuscript.

Clinical data

Policy information about clinical studies

All manuscripts should comply with the ICMJE guidelines for publication of clinical research and a completed CONSORT checklist must be included with all submissions.

Clinical trial registration	*Provide the trial registration number from ClinicalTrials.gov or an equivalent agency.*
Study protocol	*Note where the full trial protocol can be accessed OR if not available, explain why.*
Data collection	*Describe the settings and locales of data collection, noting the time periods of recruitment and data collection.*
Outcomes	*Describe how you pre-defined primary and secondary outcome measures and how you assessed these measures.*

Dual use research of concern

Policy information about dual use research of concern

Hazards

Could the accidental, deliberate or reckless misuse of agents or technologies generated in the work, or the application of information presented in the manuscript, pose a threat to:

No	Yes	
☒	☐	Public health
☒	☐	National security
☒	☐	Crops and/or livestock
☒	☐	Ecosystems
☒	☐	Any other significant area
