# Supplementary figures and images for: PDGFRA is a conserved HAND2 effector during early cardiac development
Source: Nat Cardiovasc Res. 2024 Dec 10;3(12):1531–48. doi: 10.1038/s44161-024-00574-1 (PMC11634778; doi:10.1038/s44161-024-00574-1)

14 hpf

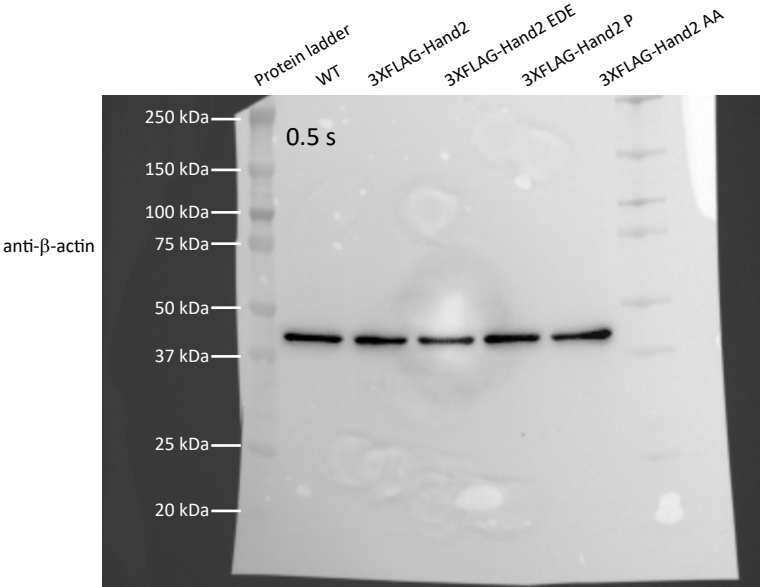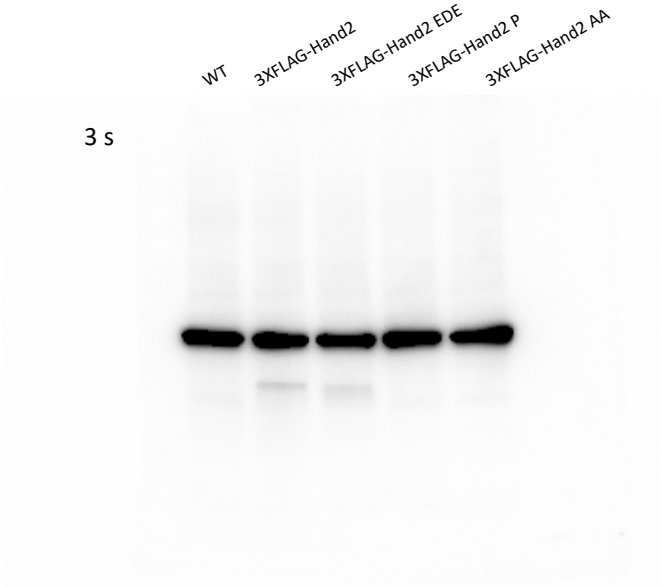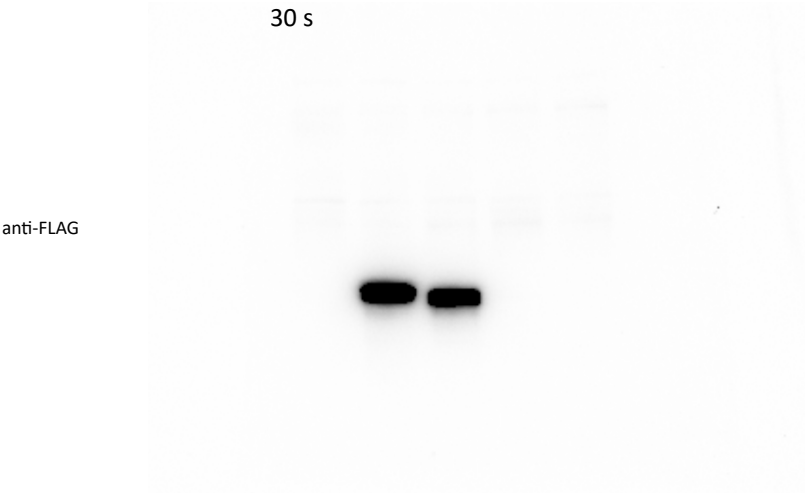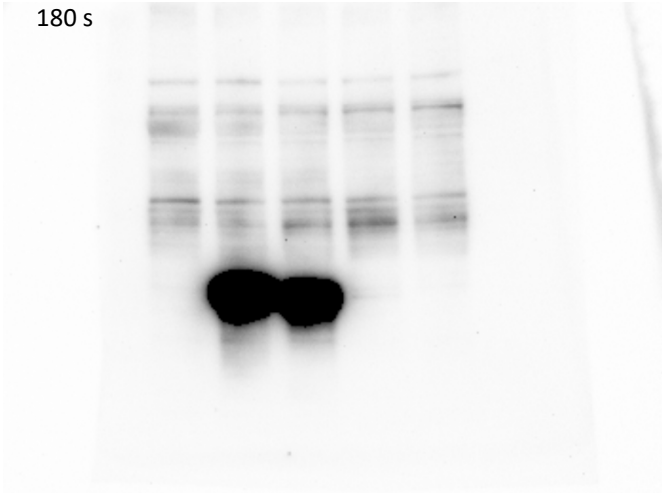

Supplement: Supplementary file 7 — Uncropped and unprocessed scans for Extended Data Fig. 5e (panel 1). [file 44161_2024_574_MOESM7_ESM.pdf]
